# Tackling Losartan Contamination: The Promise of Peroxymonosulfate/Fe(II) Advanced Oxidation Processes

**DOI:** 10.3390/molecules29102237

**Published:** 2024-05-10

**Authors:** Antonio Medici, Giovanni Luongo, Silvana Pedatella, Lucio Previtera, Giovanni Di Fabio, Armando Zarrelli

**Affiliations:** 1 Department of Chemical Sciences, University of Naples Federico II, 80126 Naples, Italy; silvana.pedatella@unina.it (S.P.); difabio@unina.it (G.D.F.); 2 Associazione Italiana per la Promozione delle Ricerche su Ambiente e Salute umana, 82030 Dugenta, Italy; giovanni.luongo@unina.it (G.L.); previter@unina.it (L.P.)

**Keywords:** losartan, peroxymonosulfate, advanced oxidation processes, degradation byproducts, water treatment

## Abstract

Losartan, an angiotensin II receptor antagonist frequently detected in wastewater effluents, poses considerable risks to both aquatic ecosystems and human health. Seeking to address this challenge, advanced oxidation processes (AOPs) emerge as robust methodologies for the efficient elimination of such contaminants. In this study, the degradation of Losartan was investigated in the presence of activated peroxymonosulfate (PMS), leveraging ferrous iron as a catalyst to enhance the oxidation process. Utilizing advanced analytical techniques such as NMR and mass spectrometry, nine distinct byproducts were characterized. Notably, seven of these byproducts were identified for the first time, providing novel insights into the degradation pathway of Losartan. The study delved into the kinetics of the degradation process, assessing the degradation efficiency attained when employing the catalyst alone versus when using it in combination with PMS. The results revealed that Losartan degradation reached a significant level of 64%, underscoring the efficacy of PMS/Fe(II) AOP techniques as promising strategies for the removal of Losartan from water systems. This research not only enriches our understanding of pollutant degradation mechanisms, but also paves the way for the development of sustainable water treatment technologies, specifically targeting the removal of pharmaceutical contaminants from aquatic environments.

## 1. Introduction

The utilization and mismanagement of substances that are not regulated by any environmental standards, whether at the state or community level, has resulted in the irreversible contamination of water bodies, soils, and air by a myriad of substances, such as personal care products [1,2], pharmaceuticals [3,4], microplastics [5,6], plant protection products [7,8], plastic additives [9,10], and even rare earth elements [11,12].

Losartan (LOS) is a medication belonging to the family of sartans, which are angiotensin II receptor antagonists [13]. Losartan treatment is primarily indicated for individuals suffering from high blood pressure and in order to protect the kidneys in hypertensive patients with type 2 diabetes who have laboratory tests showing an alteration in kidney function. Despite being one of the first of these products to be synthesized, Losartan continues to be one of the most widely used. According to a study published in 2023, there are 17 million users of angiotensin receptor blockers in the USA, UK, Canada, and Denmark, of which approximately 60% use Losartan, totalling more than 10 million users [14]. The presence of this pollutant in the environment is mainly due to direct consumption by patients. After its effects wear off, Losartan is excreted via urine and feces, reaching wastewater treatment plants. However, these facilities do not effectively reduce its presence in effluents, leading to high levels of contamination of surface waters. This occurs to the extent that that Losartan is becoming an emerging pollutant, potentially entering the food chain, and being reabsorbed via the diet. In 2007, Larsson et al. found Losartan concentrations in real plant samples from wastewater treatment that ranged from 2400 to 2500 μg/L in Patancheru, near Hyderabad, India [15]. Losartan was found in the waters of the Llobregat River (Spain), reaching a maximum concentration of 620 ng/L [16]. In 2018, Cortez et al. found Losartan in all analyzed samples, with maximum concentrations of up to 8.7 ng/L in marine samples taken from Santos Bay, Sao Paulo, Brazil. They demonstrated that even concentrations that were equivalent to environmental levels led to cyto-genotoxic effects in the gills and hemolymphs of the *Perna perna* species, showing that Losartan pollution, even at low concentrations, poses increasingly significant issues [17]. Sartans such as Irbesartan and Losartan tend to decrease in concentration following water disinfection treatments, resulting in a decrease of up to 75% for Irbesartan and 62% for Losartan [18]. This often leads to the production of more toxic degradation byproducts (**DP**) than the parent molecule [19]. These byproducts have been the subject of study in recent years, with some studies considering valsartan acid, produced by the oxidation of different sartans, as an indicator of sartan pollution [20].

To address this type of water pollution from emerging substances, advanced oxidation processes have been studied in recent years. These processes can effectively remove pollutants. They also achieve high mineralization values, allowing for the almost-complete degradation of pharmaceuticals [21,22]. Most of these methods are based on the use of less conventional oxidants like hydrogen peroxide, peroxymonosulfate, and persulfate, activating them using different conditions like temperature [23,24], microwave [25], light [26], pH [27], transition metals [28], ultrasound [29,30], and even plasma [31], to highly generate oxidant species.

In this study, we focused on isolating and characterizing the nine degradation byproducts obtained from the oxidation of Losartan using a Fenton-like process, employing peroxymonosulfate activated by Fe(II). We evaluated the kinetics of the process at different temperatures and the synergistic degradation effect in the presence of Fe(II) and PMS. Seven of the degradation products were isolated for the first time, and we hypothesized regarding the mechanisms behind their formation, with proposed for all products. We also indicated the exact structure of other potential byproducts which have not yet been isolated.

## 2. Results and Discussion

### 2.1. Degradation Experiments 

**Figure 1:**
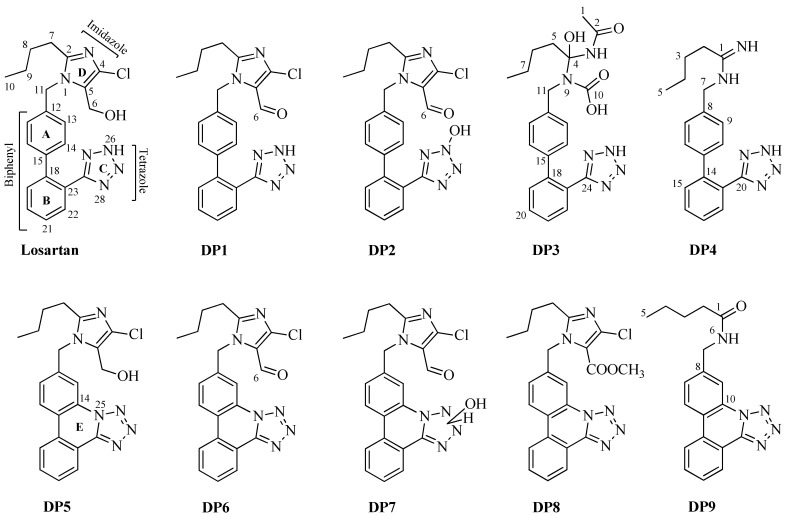
Chemical structures of Losartan and its degradation byproducts.

The experimental procedures for the oxidation of **LOS** were conducted by using conditions that ensured the complete degradation of **LOS** in both analytical and preparative settings. A solution of the drug at a concentration of 10^−5^ M was subjected to treatment for 30 min (**LOS**:PMS:Fe(II) molar ratio of 1:1:1) at room temperature. Subsequently, experiments were repeated using contaminant concentrations exceeding 10^−3^ M. We employed a significantly higher ratio of **LOS**:PMS:Fe(II) (1:24:24) to ensure the effective degradation of the target contaminant and to yield adequate quantities of degradation products (**DPs**) for subsequent structural elucidation. The resulting **DPs** (depicted in Figure 1) were purified through column chromatography and HPLC, followed by comprehensive characterization via NMR and MS analyses (see Supplementary Materials).

### 2.2. Structure Elucidation of Degradation Byproducts ***DP1***–***DP9***

In **LOS** treatment at fixed pH values, the changes in the drug were monitored via HPLC. The concentration of **DP1**–**DP9** (Figure 1) was at a maximum value after 90 min and ranged from 0.2 to 9.29%. 

**Scheme 1:**
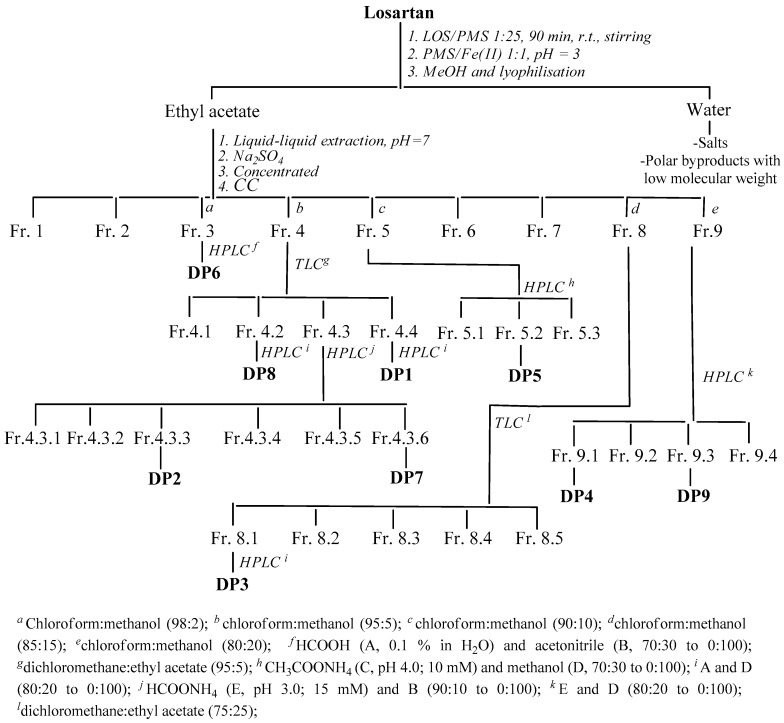
Isolation of the byproducts **DP1**–**DP7**.

**Figure 2:**
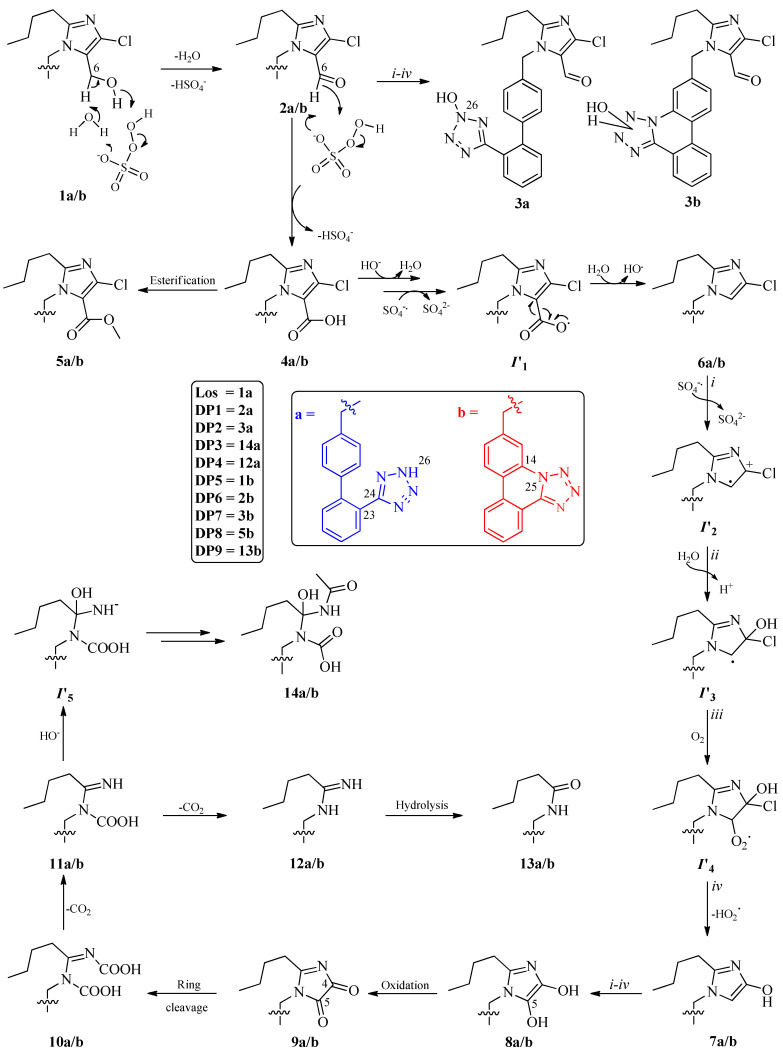
Plausible mechanism of oxidation with peroxymonosulfate.

**Figure 3:**
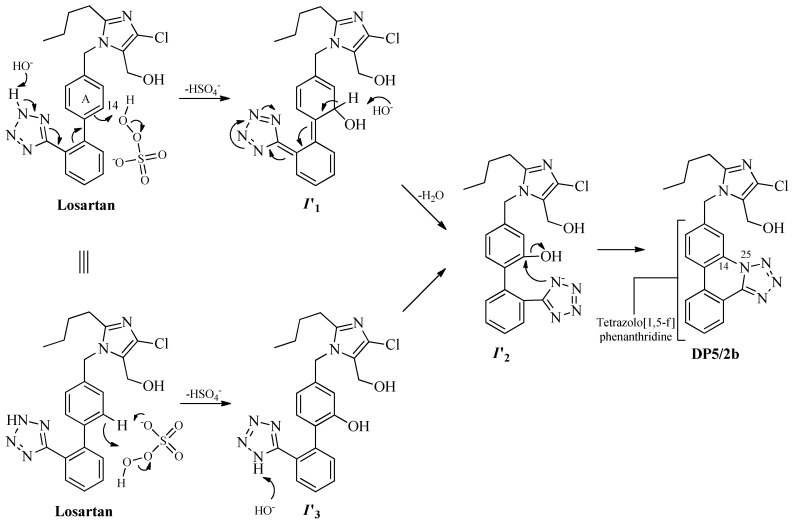
Plausible mechanisms of formation of tetrazolo [1,5-f]phenanthridine.

**DPs** were isolated using chromatographic processes (Scheme 1) and identified by employing NMR and MS analyses. The plausible mechanism of the **DPs** formation is shown in Figure 2 and Figure 3. 

As suggested by previous studies on sartans that are analogous to the compound investigated in this research, the potential degradation byproducts formed using PMS/Fe(II) show no substantial detrimental effects on algae [28]. This observation may also hold true for the byproducts identified in this study, considering their structural similarity, especially given that both belong to the sartan class.

Following the oxidation of Losartan, nine degradation products were isolated. These all retained the basic diphenyl skeleton (rings A and B) and the tetrazole cycle (ring C). Four of the nine **DPs** (**DP1**–**DP4**) showed tetrazoles that were only attached to ring B, connected through a bond between carbon C-24 of the tetrazole itself and carbon C-23 of the phenyl ring. The last 5 **DPs** (**DP5**–**DP9**) had tetrazoles that were attached to both phenyl rings A and B, forming a new six-membered cycle (ring E) due to the formation of a new bond between nitrogen N-25 of the tetrazole and carbon C-14 of aromatic ring A. The oxidative processes affected the imidazole ring D, whether partially oxidized (in **DP1**, **DP6**, and **DP8**) or opened (in **DP3**, **DP4**, and **DP9**), and the tetrazole ring C (cyclized to aromatic ring A in **DP5**, or cyclized and oxidized at one of the two positions of N-26 or N-27 in the two **DP2** and **DP7**). **DP1** (product **2a**, Figure 2) originated from the oxidation of the side chain of the imidazole ring of Losartan (product **1a**), specifically carbon C-6, with the hydroxymethyl function giving rise to the corresponding aldehyde function.

**DP2** (product **3a**) can be obtained from the previous one by the oxidation of the tetrazole ring, which is oxidized to nitrogen N-26. It should be noted that ring C can exhibit a particular type of tautomerism—reported in the scientific literature as desmotropy—which involves the possible presence of a hydrogen on each of the four different nitrogen atoms of the ring. However, considering the equivalence of the two nitrogen atoms bonded to the carbon C-24 and the free rotation of the tetrazole ring around the bond C-23–C-24, the tautomers in the liquid state are reduced to two, with the possibility that the hydrogen (and therefore the hydroxyl group in the corresponding oxidation product) is bonded to nitrogen N-25/N-28 or N-26/N-27. Generally, hydrogen is reported to be bonded to nitrogen N-26. This is because, in the solid state, this tautomer, indicated as form B, is the main one.

**DP1** (product **2a**) could then undergo oxidation at the aldehydic function (C-6) to obtain the corresponding carboxylic acid, product **4a** (Figure 2). The latter could undergo a reductive decarboxylation, known as the Kolbe reaction, to obtain product **6a**, which could then yield product **7a**, where oxidative dechlorination of the imidazole ring occurs. The latter could undergo hydroxylation at the carbon C-5 to yield product **8a**. The oxidation could then proceed at positions C-4 and C-5 (product **9a**) and further to lead to ring opening (product **10a**). An initial reductive decarboxylation would yield product **11a**, which could then form degradation **DP3** (product **14a**) or undergo a second decarboxylation to give rise to **DP4** (product **12a**).

The **DP5** (product **1b**) is the first product to exhibit the closure of a six-membered ring between the two aromatic rings and the tetrazole. We hypothesized (Figure 3) that this product could be obtained by the addition of a hydroxyl group to the carbon C-14 of ring A—forming intermediate *I*’_1_—followed by the abstraction of a proton from the same position to re-aromatize ring A and obtain a nucleophilic nitrogen on the adjacent tetrazole (intermediate *I*’_2_). The final step would involve the closure of the cycle via the nucleophilic attack of the nitrogen N-25 of the tetrazole on the carbon C-14 of the adjacent aromatic ring. 

Alternatively, one could hypothesize the oxidation of ring A at carbon C-14, followed by the abstraction of a proton from the nitrogen N-25 of the tetrazole. The intramolecular nucleophilic substitution would then explain the formation of the degradation product, **DP5**.

The degradation product known as **DP5** (product **1b**) can be considered the precursor to the last four degradation products obtained (**DP6**–**DP9**). Specifically, **DP6** (product **2b**) is the corresponding aldehyde of **DP5**, obtained by oxidizing the hydroxymethyl group at C-6. In turn, the oxidation of **DP6** on the tetrazole ring, with hydroxyl group oxidation at nitrogen N-26 (or N-27), would explain the formation of **DP7** (product **3b**), while the oxidation of carbon C-6 in **DP6** to the corresponding carboxylic acid **4b** and its subsequent methylation would yield the corresponding ester, **DP8** (product **5b**).

Finally, the reductive decarboxylation of product **4b** and the subsequent oxidation and opening of the imidazole ring would account for the formation of product **DP9** (product **13b**), corresponding to the hydrolysis product of **DP4** (product **12a**) released with the additional formation of cycle E. 

**Figure 4:**
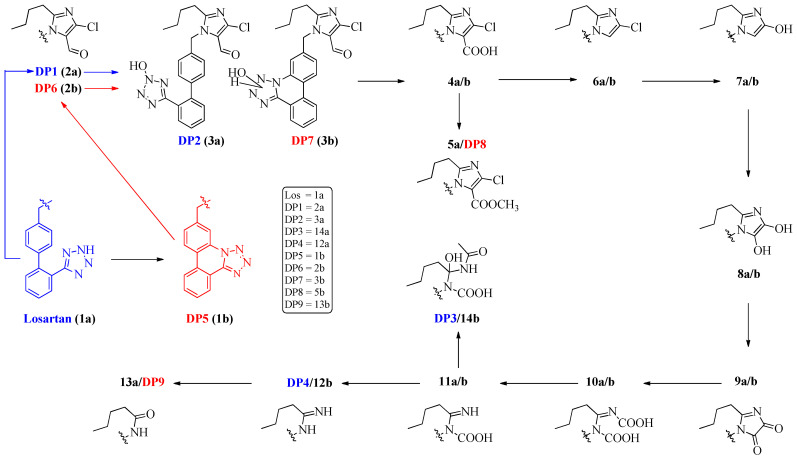
Degradation products isolated and those that could form but have not yet been isolated.

Observing the nine identified **DPs** resulting from the oxidation reaction with peroxymonosulfate, two main structural modifications are noticeable: (a) the oxidation of the hydroxymethyl side chain of the imidazole ring (**DP1** and **DP6**); (b) the formation of an intramolecular bond and the closure of ring E (**DP5**–**DP9**). Thus, degradation **DP1** can be considered the precursor to obtaining **DP2**–**DP4**, while **DP5** can be considered to correspond to **DP1** and to be the precursor **for** obtaining **DP6**–**DP9**. Similarly, it is straightforward to consider **DP2** and **DP7** as corresponding, differing only in the absence or presence of the hydroxylated ring E. Likewise, products **DP4** and **DP9** can be considered to correspond, with the latter featuring the ring E and a carbonyl function instead of an iminium one. In general, starting from **DP1** and **DP5**, the generation of other degradation products can be hypothesized based on the oxidation of carbon C-6 and the subsequent degradation of ring D, as summarized in Figure 4. It is conceivable that other products may have formed, albeit in lesser quantities, that have not yet been identified or degraded more rapidly than others, which could confirm the proposed mechanism.

### 2.3. Spectral Data

Losartan: (1-((2′-(2H-Tetrazol-5-yl)-[1,1′-biphenyl]-4-yl)methyl)-2-butyl-4-chloro-1H -imi dazol-5-yl)methanol. White powder. NMR data see Table S1. MS-TOF (positive ions): *m*/*z* calculated for C_22_H_23_ClN_6_O 422.16 [M]^+^; found 423.17 [M + H]^+^ (84%), 425.17 (26%), 424.18 (21%), 426.17 (7%). 

**DP1**: 1-((2′-(2H-Tetrazol-5-yl)-[1,1′-biphenyl]-4-yl)methyl)-2-butyl-4-chloro-1H-imidazole-5-carbaldehyde. Gray powder. NMR data see Table S2. MS-TOF (negative ions): *m*/*z* calculated for C_22_H_21_ClN_6_O 420.15 [M]^+^; found 419.15 [M − H]^−^ (90%), 420.16 (20%), 421.15 (30%), 422.15 (7%).

**DP2**: 2-butyl-4-chloro-1-((2′-(2-hydroxy-2H-tetrazol-5-yl)-[1,1′-biphenyl]-4-yl)methyl)-1H-imidazole-5-carbaldehyde. Gray powder. NMR data see Table S3. MS-TOF (positive ions): *m*/*z* calculated for C_22_H_21_ClN_6_O_2_ 436.14 [M]^+^; found 437.15 [M + H]^+^ (88%), 439.14 (28%), 438.15 (24%), 440.17 (6%).

**DP3**: ((2′-(2H-Tetrazol-5-yl)-[1,1′-biphenyl]-4-yl)methyl)(1-acetamido-1-hydroxypentyl) carbamic acid. Gray powder. NMR data see Table S4. MS-TOF (positive ions): *m*/*z* calculated for C_22_H_26_N_6_O_4_ 438.20 [M]^+^; found 461.21 [M + Na]^+^ (48%), 462.21 (10%). 

**DP4**: N-((2′-(2H-Tetrazol-5-yl)-[1,1′-biphenyl]-4-yl)methyl)pentanimidamide. White powder. NMR data see Table S5. MS-TOF (positive ions): *m*/*z* calculated for C_19_H_22_N_6_ 334.19 [M]^+^; found 335.20 [M + H]^+^ (90%), 336.20 (22%).

**DP5**: (2-Butyl-4-chloro-1-(tetrazolo [1,5-f]phenanthridin-6-ylmethyl)-1H-imidazol-5-yl) methanol. Gray powder. NMR data see Table S6. MS-TOF (positive ions): *m*/*z* calculated for C_22_H_21_ClN_6_O 420.15 [M]^+^; found 421.16 [M + H]^+^ (85%), 423.16 (24.0%), 422.16 (18%). 

**DP6**: 2-Butyl-4-chloro-1-(tetrazolo [1,5-f]phenanthridin-6-ylmethyl)-1H-imidazole-5-carbaldehyde. Gray powder. NMR data see Table S7. MS-TOF (positive ions): *m*/*z* calculated for C_22_H_19_ClN_6_O 418.13 [M]^+^; found 419.13 [M + H]^+^ (100%), 421.14 (30%), 420.14 (24%). 

**DP7**: 2-Butyl-4-chloro-1-((2-hydroxy-2,3-dihydrotetrazolo [1,5-f]phenanthridin-6-yl)meth yl)-1H-imidazole-5-carbaldehyde. Gray powder. NMR data see Table S8. MS-TOF (positive ions): *m*/*z* calculated for C_22_H_21_ClN_6_O_2_ 436.14 [M]^+^; found 437.15 [M + H]^+^ (95%), 439.16 (31%), 438.17 (22%), 440.15 (5%). 

**DP8**: Methyl 2-butyl-4-chloro-1-(tetrazolo [1,5-f]phenanthridin-6-ylmethyl)-1H-imidazole -5-carboxylate. Gray powder. NMR data see Table S9. MS-TOF (positive ions): *m*/*z* calculated for C_23_H_21_ClN_6_O_2_ 448.14 [M]^+^; found 449.15 [M + H]^+^ (100%), 451.15 (32%), 450.16 (25%), 452.16 (7%).

**DP9**: N-(Tetrazolo [1,5-f]phenanthridin-6-ylmethyl)pentanamide. Gray powder. NMR data see Table S10. MS-TOF (positive ions): *m*/*z* calculated for C_19_H_19_N_5_O 333.16 [M]^+^; found 334.16 [M + H]^+^ (87%), 335.17 (21%).

### 2.4. Kinetics of Losartan Degradation in PMS/Fe(II) System

**Figure 5:**
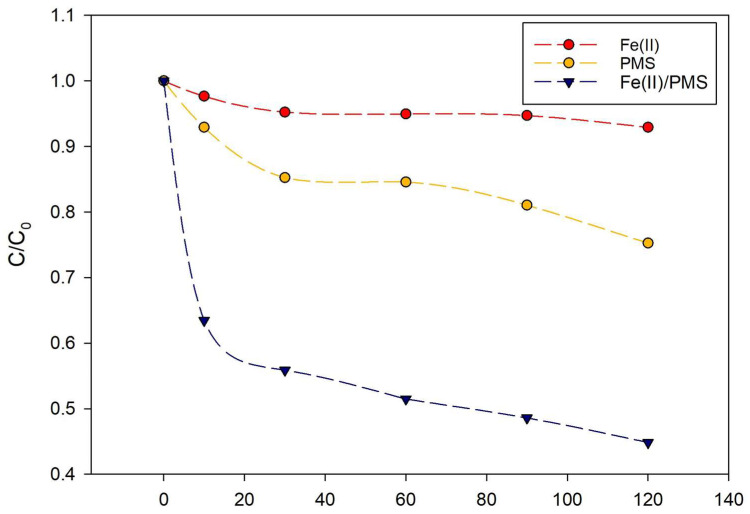
Different [**LOS**] = 2.14 mM removal in presence of [Fe(II)] = 52 mM and/or [PMS] = 52 mM.

To comprehensively investigate the oxidation process of **LOS** in the presence of the PMS/Fe(II) system, we initially examined the stability of the pollutant at pH 3 in the presence of Fe(II) sulfate heptahydrate, which resulted in the removal of only 5% of the pollutant (Figure 5). Subsequently, another experiment was conducted to assess the degradation directly attributable to the oxidant PMS, which was compared with the degradation observed in the presence of only Fe(II). In this experiment, the degradation halted at 20%. This highlights the difference between the degradation in the presence of iron and that when the system is solely in the presence of PMS.

As shown in Figure 5, a synergistic effect arises from oxidation in the presence of the Fe(II) catalyst, which is likely due to the rapid reaction between Fe(II) and PMS, yielding highly reactive radical species such as SO_4_^−^. These radical species consume Fe(II) to form Fe(III) (Equation (1)), while the reaction between Fe(III) and PMS is a slower process, leading to the formation of less reactive radicals (Equation (2)) [32]. Nonetheless, the generation of Fe(II) allows for the continuation of the reaction (Equation (1)).

(1) $$Fe\left(II\right)+HSO_{5}^{-}\to Fe\left(III\right)+SO_{4}^{-}.$$

(2) $$Fe\left(III\right)+HSO_{5}^{-}\to Fe\left(II\right)+SO_{5}^{-}.$$

**Figure 6:**
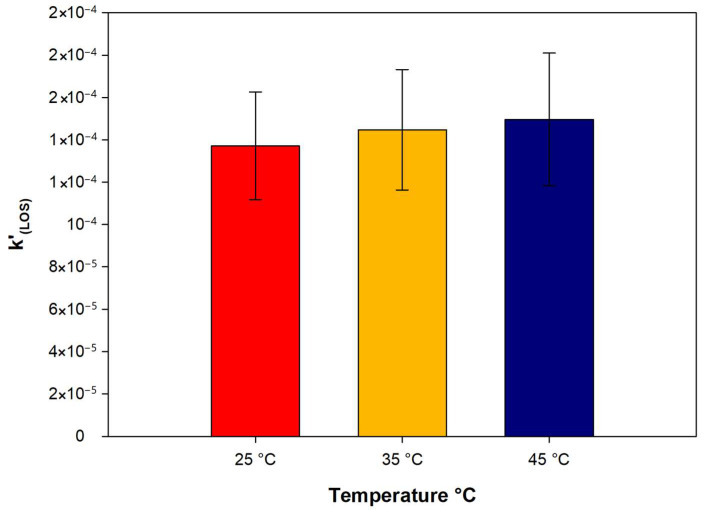
Pseudo-first-order rate constant for $k_{LOS}^{\prime }$, at different temperature.

Further studies were conducted to evaluate the degradation of Losartan as a function of temperature, conducting trials at 25 °C, 35 °C, and 45 °C (Figure 6). As anticipated, increasing the temperature resulted in a higher rate constant for $k_{LOS}^{\prime }$, calculated as pseudo-first-order decay using Equation (3):

(3) $$ln\frac{\left[LOS\right]}{{\left[LOS\right]}_{0}}=-k_{LOS}^{\prime }t$$

where [***LOS***]_0_ and [***LOS***] represent the initial and residual concentrations of **LOS** at time *t*, respectively, and $k_{LOS}^{\prime }$ is the pseudo-first-order apparent rate constant in s^−1^.

## 3. Materials and Methods

### 3.1. Drug and Reagents

Losartan potassium (98%), OXONE^®^ (triple-salt KHSO_5_ * 0.5KHSO_4_ * 0.5K_2_SO_4_, of which the oxidizing species is KHSO_5_, called potassium monopersulfate or potassium peroxymonosulfate/PMS) and iron (II) sulfate heptahydrate (FeSO_4_ * 7H_2_O) were purchased from Merck (Darmstadt, Germany). Solvents were purchased from Merck (Darmstadt, Germany), were of an HPLC grade, and were used as received. All other chemicals were of an analytical grade and were supplied by Merck, Darmstadt, Germany.

### 3.2. Peroxymonosulfate Reaction

#### 3.2.1. Apparatus and Equipment

Kieselgel 60 (230–400 mesh, Merck, Darmstadt, Germany) was used for column chromatography (CC). HPLC analysis utilized a Shimadzu LC-8A system, equipped with a Shimadzu SPD-10A VP UV-vis detector (Shimadzu, Milan, Italy). NMR spectra (^1^H and ^13^C) were recorded at 400 MHz and 25 °C (Bruker DRX, Bruker Avance), and results were referenced to residual solvent signals (CDCl_3_, δ_H_ 7.27 and δ_C_ 77.0; CD_3_OD, δ_H_ 3.30 and δ_C_ 49.0). Proton-detected heteronuclear correlations were measured using gradient heteronuclear single-quantum coherence (HSQC), optimized for ^1^J_HC_ = 155 Hz, and gradient heteronuclear multiple-bond correlation (HMBC), optimized for ^n^J_HC_ = 8 Hz. MALDI-TOF mass spectrometric analyses were conducted on a Voyager-De Pro MALDI mass-spectrometer (PerSeptive Biosystems, Framingham, MA, USA). UV-vis analysis was performed on a Lambda 12 UV-vis spectrophotometer (Perkin Elmer, Waltham, MA, USA). The lyophilization of samples was performed using a LyovaporTM-200 (Buchi, Cornaredo, Italy), with a compressor featuring a cooling capacity of 1.97 kW for 50 Hz and a minimum condenser temperature of −55 °C.

#### 3.2.2. Peroxymonosulfate-Based Advanced Oxidation Reaction

A solution of **LOS** at a concentration of 10^−5^ M was treated with Fe(II)/OXONE^®^ (molar ratio of **LOS**/Fe(II)/OXONE^®^ 1:1:1) for 30 min at room temperature at pH 3.0, as pH plays a crucial role in the presence of Fe(II) metal ions in homogeneous phases during reactions [33]. The presence of **LOS** was determined spectrophotometrically, with absorbance peaks measured at 254 nm. The absorbance values were then converted into concentrations using a calibration curve prepared from standard solutions with known **LOS** concentrations. Although **DP1**–**DP14** (Figure 1) were formed under these conditions, their abundance was too low for isolation. However, their retention times were compared with those of the byproducts obtained from the degradation of **LOS** in the preparative experiments described subsequently. **DPs** that are common to both the analytical and preparative experiments were then isolated from the ethyl acetate extract of the aqueous solution.

Preparative experiments were conducted using a solution of **LOS** at a concentration higher than 10^−3^ M. Specifically, 495 mg (1.07 mmol) of **LOS** dissolved in water was added to a final volume of 0.5 L. OXONE^®^ (8.20 g, 26 mmol, molar ratio LOS/OXONE^®^ 1:24) under magnetic stirring at room temperature, followed by the addition of iron (II) sulfate heptahydrate (7.50 g, 26 mmol, molar ratio Fe(II)/OXONE^®^ 1:1). The pH of the solution was adjusted to 3.0 with H_2_SO_4_ 1 M. The reaction progress was monitored by sampling the solution every 15 min, quenching with excess methanol, and drying under vacuum conditions. The resulting residue was dissolved in a saturated sodium bicarbonate solution, extracted with ethyl acetate, and analyzed via HPLC. The reaction was terminated after 2 h to ensure the maximal degradation of **LOS** and the formation of its **DPs**. This was performed by quenching with excess methanol and concentrating via lyophilization. The residue was dissolved in water and pH-adjusted to 7.0 before extraction with ethyl acetate. The extraction yielded an organic fraction of 467 mg, which was chromatographed to obtain the **DPs**.

#### 3.2.3. Product Isolation Procedure

The organic fraction obtained from the preparative experiment (467 mg) was chromatographed with a gradient of chloroform/methanol (100:0 to 80:20) on silica gel to yield 9 fractions. Fraction 3 (Fr. 3), obtained from elution with chloroform/methanol 98:2, contained **DP6** (19 mg). This was further purified using HPLC under gradient condition f of HCOOH (0.1% in H_2_O) and acetonitrile (70:30 to 0:100) on a Phenomenex Luna 5 μm C18 100 Å (250 × 10.00 mm) HPLC column at a solvent flow rate of 5 mL/min. Fraction 4 (Fr. 4), obtained from elution with chloroform/methanol 95:5, was subsequently purified via preparative thin-layer chromatography (TLC) and eluted with dichloromethane–ethyl acetate 95:5, resulting in 4 subfractions. The **DP8** (2 mg) and **DP1** (10 mg) were obtained from subfractions Fr. 4.2 and Fr. 4.4, respectively, and these were purified using the same gradient i in HPLC, namely HCOOH (0.1% in H_2_O) and methanol (80:20 to 0:100). Fraction 4.3 (Fr. 4.3) was purified via HPLC using gradient j, involving HCOONH_4_ (pH 3.0; 15 mM) and acetonitrile (90:10 to 0:100). This was performed on a Phenomenex Kromasil 10 μm 100 Å C18 (250 × 10.00 mm) HPLC column at a solvent flow rate of 5 mL/min, resulting in **DP2** (25 mg) and **DP7** (12 mg). Fraction 5 (Fr. 5), obtained from elution with chloroform/methanol 90:10, was purified via HPLC under gradient conditions h, entailing CH_3_COONH_4_ (pH 4.0; 10 mM) and methanol (70:30 to 0:100). This was performed using a Phenomenex Prodigy 10 μm ODS (250 × 10.00 mm) HPLC column at a solvent flow rate of 5 mL/min, yielding **DP5** (18 mg). Fraction 8 (Fr. 8), obtained from elution with chloroform/methanol 85:15, was purified via preparative TLC under condition l, entailing dichloromethane–ethyl acetate (80:20), which resulted in 5 subfractions. Subsequently, subfraction 8.1 (Fr. 8.1) was further purified via HPLC under gradient condition i, entailing HCOOH (0.1% in H_2_O) and methanol (80:20 to 0:100). We used a Phenomenex Synergi 10 μm 110 Å C18 (250 × 10.00 mm) column at a solvent flow rate of 5 mL/min, yielding **DP3** (46 mg). Finally, fraction 9 (Fr. 9), obtained from elution with chloroform/methanol 80:20, was purified via HPLC under the following gradient conditions k: HCOONH_4_, (pH 3.0; 15 mM) and methanol (80:20 to 0:100). We used the same column as that used for Fr. 5, yielding **DP4** (1 mg) and **DP9** (1 mg).

## 4. Conclusions

In conclusion, our study sheds light on the concerning presence and potential risks associated with Losartan contamination in aquatic environments. The widespread occurrence of Losartan in surface waters highlights the urgent need to develop effective water treatment strategies to mitigate its impact on ecosystems and human health. Our findings demonstrate the capability of advanced oxidation processes, such as Fe(II)-activated peroxymonosulfate, to degrade Losartan and potentially other emerging contaminants, offering a promising solution for water treatment. Nine degradation products were thus isolated and structurally determined via NMR and mass studies. These degradation byproducts, obtained in percentages between 0.20 and 9.29% and for a total yield of 27%, compared to a complete mineralization of the starting product equal to 64%, provide valuable insights into the transformation pathways and environmental fate of Losartan and structurally similar micropollutants. These insights contribute to our understanding of the effectiveness of oxidation processes in terms of removing pharmaceutical contaminants from water sources.

## Data Availability

Data are contained within the article and Supplementary Materials.

## Supplementary Materials

The following supporting information can be downloaded at: https://www.mdpi.com/article/10.3390/molecules29102237/s1.

## References

[1] Osuoha J.O., Anyanwu B.O., Ejileugha C. (2023). Pharmaceuticals and Personal Care Products as Emerging Contaminants: Need for Combined Treatment Strategy. J. Hazard. Mater. Adv..

[2] Khalid M., Abdollahi M. (2021). Environmental Distribution of Personal Care Products and Their Effects on Human Health. Iran J. Pharm. Res..

[3] Pot E.J., Milakovic M., Chaumot A., Seidensticker S., Melling M., Supriatin A., Sherif S. (2022). Pharmaceutical Pollution of the World’s Rivers. Proc. Natl. Acad. Sci. USA.

[4] Larsson D.G.J. (2014). Pollution from Drug Manufacturing: Review and Perspectives. Philos. Trans. R. Soc. Lond. B Biol. Sci..

[5] Wu W.M., Yang J., Criddle C.S. (2017). Microplastics Pollution and Reduction Strategies. Front. Environ. Sci. Eng..

[6] Rezania S., Park J., Md Din M.F., Mat Taib S., Talaiekhozani A., Kumar Yadav K., Kamyab H. (2018). Microplastics Pollution in Different Aquatic Environments and Biota: A Review of Recent Studies. Mar. Pollut. Bull..

[7] Hassaan M.A., El Nemr A. (2020). Pesticides Pollution: Classifications, Human Health Impact, Extraction and Treatment Techniques. Egypt. Aquat. Res..

[8] Mali H., Shah C., Raghunandan B.H., Prajapati A.S., Patel D.H., Trivedi U., Subramanian R.B. (2023). Organophosphate Pesticides an Emerging Environmental Contaminant: Pollution, Toxicity, Bioremediation Progress, and Remaining Challenges. J. Environ. Sci..

[9] Lin Z., Wang L., Jia Y., Zhang Y., Dong Q., Huang C. (2017). A Study on Environmental Bisphenol A Pollution in Plastics Industry Areas. Water Air Soil Pollut..

[10] Fu P., Kawamura K. (2010). Ubiquity of Bisphenol A in the Atmosphere. Environ. Pollut..

[11] Trifuoggi M., Donadio C., Ferrara L., Stanislao C., Toscanesi M., Arienzo M. (2018). Levels of Pollution of Rare Earth Elements in the Surface Sediments from the Gulf of Pozzuoli (Campania, Italy). Mar. Pollut. Bull..

[12] Ramos S.J., Dinali G.S., Oliveira C., Martins G.C., Moreira C.G., Siqueira J.O., Guilherme L.R.G. (2016). Rare Earth Elements in the Soil Environment. Curr. Pollut. Rep..

[13] Timmermans P.B., Wong P.C., Chiu A.T., Herblin W.F., Benfield P., Carini D.J., Lee R.J., Wexler R.R., Anne Saye J.M., Smith R.D. (1993). Angiotensin II Receptors and Angiotensin II Receptor Antagonists. Pharmacol. Rev..

[14] Eworuke E., Shinde M., Hou L., Paterson M.J., Jensen P.B., Maro J.C., Rai A., Scarnecchia D., Pennap D., Woronow D. (2023). Valsartan, Losartan and Irbesartan Use in the USA, UK, Canada and Denmark after the Nitrosamine Recalls: A Descriptive Cohort Study. BMJ Open.

[15] Larsson D.G.J., de Pedro C., Paxeus N. (2007). Effluent from Drug Manufactures Contains Extremely High Levels of Pharmaceuticals. J. Hazard. Mater..

[16] Huerta-Fontela M., Galceran M.T., Ventura F. (2011). Occurrence and Removal of Pharmaceuticals and Hormones through Drinking Water Treatment. Water Res..

[17] Cortez F.S., da Silva Souza L., Guimarães L.L., Almeida J.E., Pusceddu F.H., Maranho L.A., Mota L.G., Nobre C.R., Moreno B.B., Abessa D.M.d.S. (2018). Ecotoxicological Effects of Losartan on the Brown Mussel Perna Perna and Its Occurrence in Seawater from Santos Bay (Brazil). Sci. Total Environ..

[18] Ladhari A., La Mura G., Di Marino C., Di Fabio G., Zarrelli A. (2021). Sartans: What They Are for, How They Degrade, Where They Are Found and How They Transform. Sustain. Chem. Pharm..

[19] Siciliano A., Medici A., Guida M., Libralato G., Saviano L., Previtera L., Di Fabio G., Zarrelli A. (2023). Newly Discovered Irbesartan Disinfection Byproducts via Chlorination: Investigating Potential Environmental Toxicity. Appl. Sci..

[20] Nödler K., Hillebrand O., Idzik K., Strathmann M., Schiperski F., Zirlewagen J., Licha T. (2013). Occurrence and Fate of the Angiotensin II Receptor Antagonist Transformation Product Valsartan Acid in the Water Cycle—A Comparative Study with Selected β-Blockers and the Persistent Anthropogenic Wastewater Indicators Carbamazepine and Acesulfame. Water Res..

[21] Kanakaraju D., Glass B.D., Oelgemöller M. (2018). Advanced Oxidation Process-Mediated Removal of Pharmaceuticals from Water: A Review. J. Environ. Manag..

[22] Deng Y., Zhao R. (2015). Advanced Oxidation Processes (AOPs) in Wastewater Treatment. Curr. Pollut. Rep..

[23] Zrinyi N., Pham A.L.T. (2017). Oxidation of Benzoic Acid by Heat-Activated Persulfate: Effect of Temperature on Transformation Pathway and Product Distribution. Water Res..

[24] Ma J., Li H., Chi L., Chen H., Chen C. (2017). Changes in Activation Energy and Kinetics of Heat-Activated Persulfate Oxidation of Phenol in Response to Changes in PH and Temperature. Chemosphere.

[25] Yang S., Wang P., Yang X., Wei G., Zhang W., Shan L. (2009). A Novel Advanced Oxidation Process to Degrade Organic Pollutants in Wastewater: Microwave-Activated Persulfate Oxidation. J. Environ. Sci..

[26] Gabet A., Métivier H., de Brauer C., Mailhot G., Brigante M. (2021). Hydrogen Peroxide and Persulfate Activation Using UVA-UVB Radiation: Degradation of Estrogenic Compounds and Application in Sewage Treatment Plant Waters. J. Hazard. Mater..

[27] Wacławek S., Lutze H.V., Sharma V.K., Xiao R., Dionysiou D.D. (2022). Revisit the Alkaline Activation of Peroxydisulfate and Peroxymonosulfate. Curr. Opin. Chem. Eng..

[28] Medici A., Lavorgna M., Isidori M., Russo C., Orlo E., Luongo G., Di Fabio G., Zarrelli A. (2024). Advanced Oxidation Process of Valsartan by Activated Peroxymonosulfate: Chemical Characterization and Ecotoxicological Effects of Its Byproducts. Sci. Total Environ..

[29] Rayaroth M.P., Aravind U.K., Aravindakumar C.T. (2017). Ultrasound Based AOP for Emerging Pollutants: From Degradation to Mechanism. Environ. Sci. Pollut. Res..

[30] Joseph C.G., Li Puma G., Bono A., Krishnaiah D. (2009). Sonophotocatalysis in Advanced Oxidation Process: A Short Review. Ultrason. Sonochem..

[31] Verdini F., Calcio Gaudino E., Canova E., Colia M.C., Cravotto G. (2023). Highly Efficient Tetracycline Degradation under Simultaneous Hydrodynamic Cavitation and Electrical Discharge Plasma in Flow. Ind. Eng. Chem. Res..

[32] Xiao S., Cheng M., Zhong H., Liu Z., Liu Y., Yang X., Liang Q. (2020). Iron-mediated activation of persulfate and peroxymonosulfate in both homogeneous and heterogeneous ways: A review. Chem. Eng. J..

[33] Ji Y., Wang L., Jiang M., Yang Y., Yang P., Lu J., Ferronato C.C., Chovelon J.M. (2017). Ferrous-activated peroxymonosulfate oxidation of antimicrobial agent sulfaquinoxaline and structurally related compounds in aqueous solution: Kinetics, products, and transformation pathways. Environ. Sci. Pollut. Res..

