# Supplementary material for: Tackling Losartan Contamination: The Promise of Peroxymonosulfate/Fe(II) Advanced Oxidation Processes
Source: Molecules. 2024 May 10;29(10):2237. doi: 10.3390/molecules29102237 (PMC11123791; doi:10.3390/molecules29102237)
Supplement: Supplementary file 1 [file molecules-29-02237-s001.zip › molecules-2984723-supplementary.pdf]

Table S1. Data obtained from the mono- and two-dimensional NMR analysis of Losartan, in deuterated methanol (CD_3OH).

**Position**	**Residue**	**$^{13}C^a$**	**$^1H^a$, multiplicity (J in Hz)**	**^1H-^1H COSY**	**^1H-^{13}C HMBC**
2	C	150.01	-		
4	C	127.39	-		
5	C	126.85	-		
6	CH_2	53.01	4.43, s		127.39, 126.85
7	CH_2	27.40	2.57, t (7.9)	1.53	150.01, 30.95, 23.24
8	CH_2	30.95	1.53, quint (7.9)	2.57, 1.31	150.01, 23.24, 14.03
9	CH_2	23.24	1.31, sest (7.6)	1.53, 0.86	30.95, 27.40, 14.03
10	CH_3	14.03	0.86, t (7.6)	1.31	30.95, 23.24
11	CH_2	48.29	5.25, s		150.01, 136.08, 126.85, 126.70
12	C	136.08	-		
13/17	CH	126.70	6.91, d (8.3)	7.11	142.24, 126.70, 48.29
14/18	CH	130.84	7.11, d (8.3)	6.91	142.62, 136.08, 130.84
15	C	142.24	-		
18	C	142.62	-		
19	CH	131.17	7.45, dd (7.0, 1.6)	7.49, 7.43	142.24, 131.11, 128.19
20	CH	130.02	7.49, t (7.1)	7.45, 7.43	142.62, 131.82
21	CH	128.19	7.43, t (7.1)	7.53, 7.49	131.17, 131.11
22	CH	131.82	7.53, dd (7.0, 1.8)	7.49, 7.43	162.71, 142.62, 130.02
23	C	131.11	-		
24	C	162.71	-		

[a]Chemical shifts in ppm.

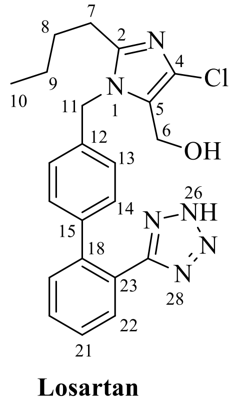

Losartan

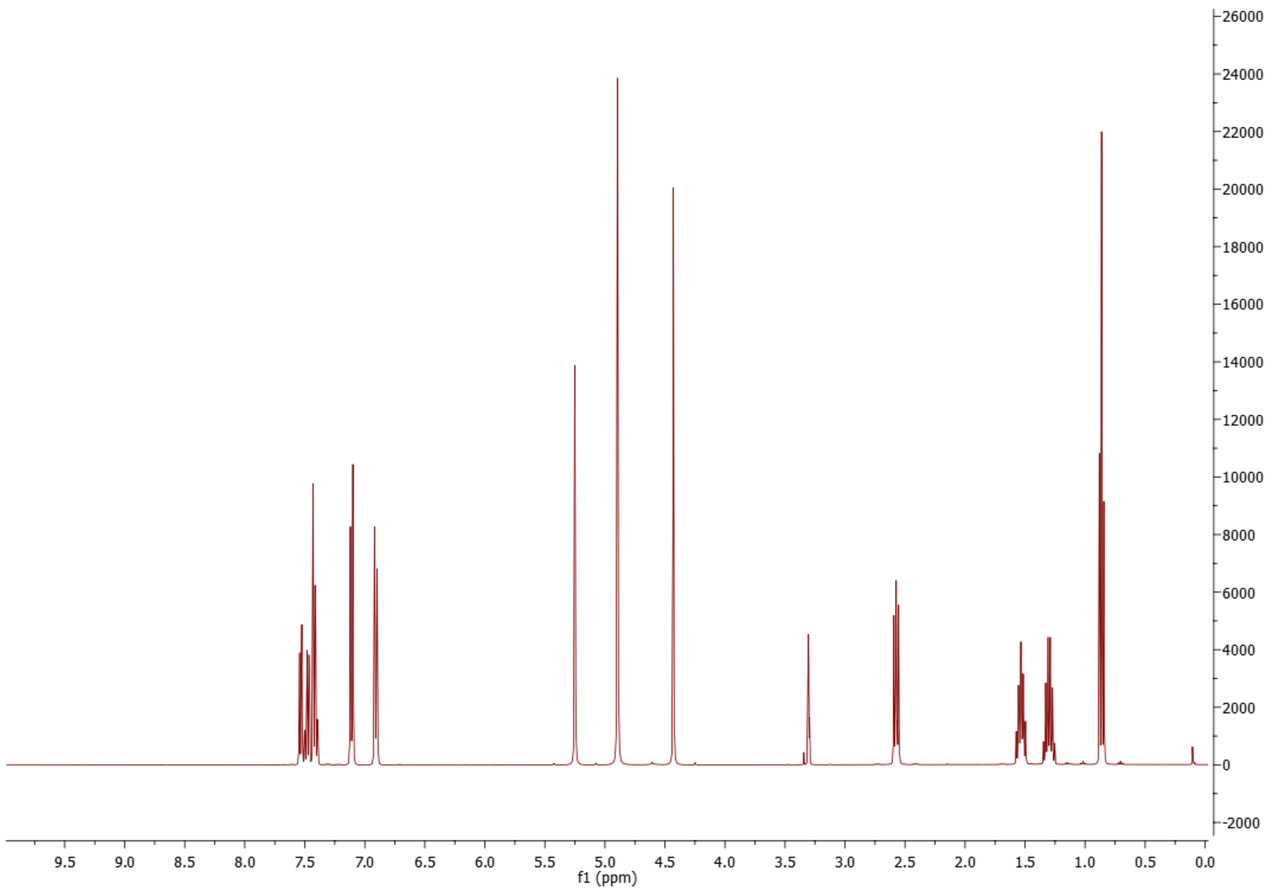
26000
24000
22000
20000
18000
16000
14000
12000
10000
8000
6000
4000
2000
0
-2000
9.5
9.0
8.5
8.0
7.5
7.0
6.5
6.0
5.5
5.0
4.5
4.0
3.5
3.0
2.5
2.0
1.5
1.0
0.5
0.0
f1 (ppm)

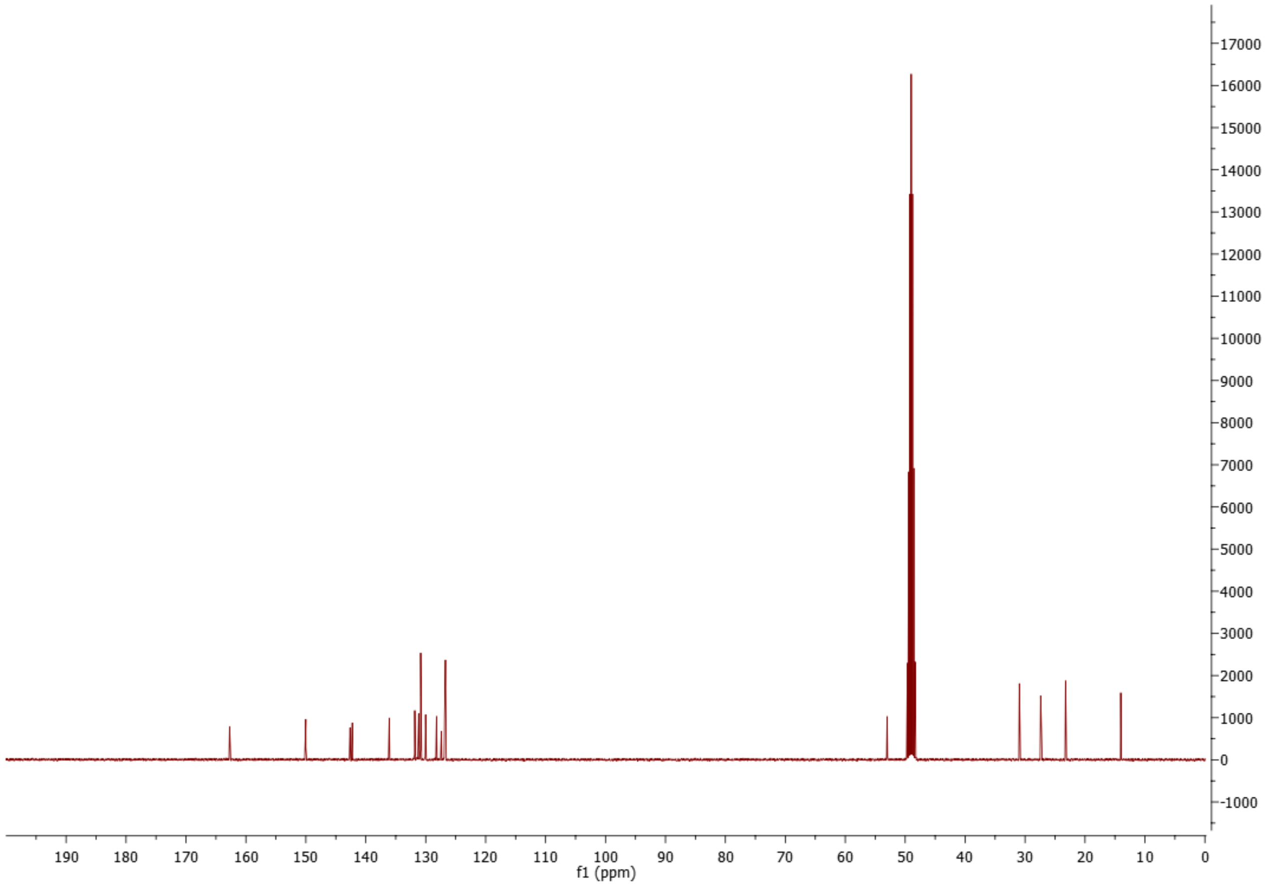
17000
16000
15000
14000
13000
12000
11000
10000
9000
8000
7000
6000
5000
4000
3000
2000
1000
0
-1000
190
180
170
160
150
140
130
120
110
100
90
80
70
60
50
40
30
20
10
0
f1 (ppm)

Table S2. Data obtained from the mono- and two-dimensional NMR analysis of DP1, in deuterated chloroform ($CDCl_3$).

**Position**	**Residue**	**$^{13}C^{a}$**	**$^{1}H^{a}$, multiplicity (J in Hz)**	**^{1}H-^{1}H COSY**	**^{1}H-^{13}C HMBC**
2	C	154.80	-		
4	C	143.23	-		
5	C	124.19	-		
6	CH	177.99	9.55, s		143.23, 124.19
7	CH_2	26.39	2.59, t (7.2)	1.62	154.80, 29.22, 22.30
8	CH_2	29.22	1.62, quint (7.2)	2.59, 1.30	154.80, 29.22, 22.30, 13.61
9	CH_2	22.30	1.30, sest (7.2)	1.62, 0.85	29.22, 26.39, 13.61
10	CH_3	13.61	0.85, t (7.3)	1.30	29.22, 22.30
11	CH_2	47.94	5.49, s		154.80, 126.73, 124.19
12	C	135.42	-		
13/17	CH	126.73	6.97, d (8.1)	7.09	139.17, 126.73, 47.94
14/18	CH	129.57	7.09, d (8.1)	6.97	140.58, 139.17, 135.42, 129.57
15	C	139.17	-		
18	C	140.58	-		
19	CH	130.74	7.40, dd (7.7, 1.2)	7.58, 7.50	139.17, 128.30, 122.65
20	CH	131.32	7.58, dt (7.6, 1.3)	7.50, 7.40	140.58, 130.74
21	CH	128.30	7.50, dt (7.6, 1.4)	7.89, 7.58	130.74, 122.65
22	CH	130.74	7.89, dd (7.7, 1.1)	7.58, 7.50	154.85, 140.58, 131.32
23	C	122.65	-		
24	C	154.85	-		

[a]Chemical shifts in ppm.

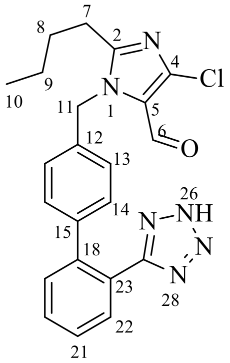

DP1

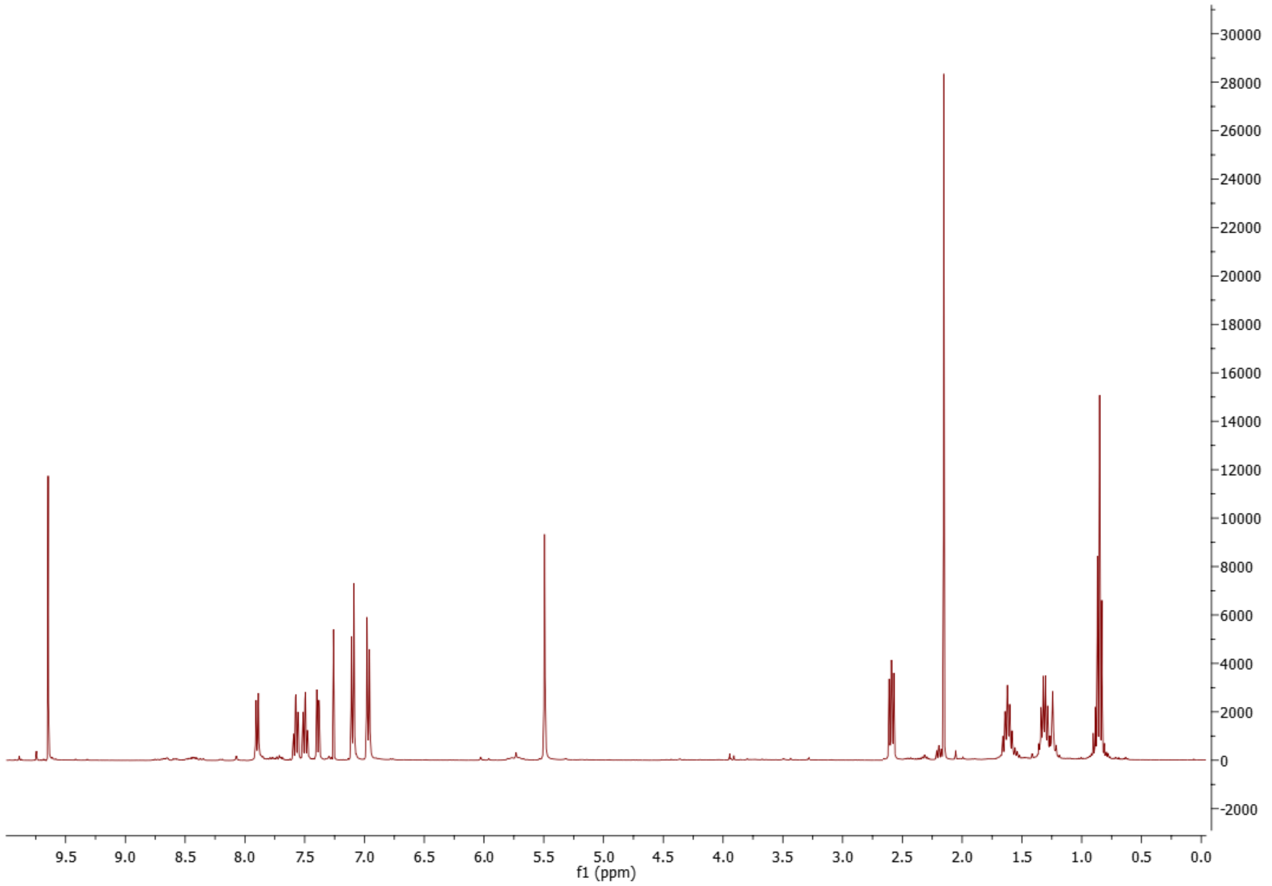
30000
28000
26000
24000
22000
20000
18000
16000
14000
12000
10000
8000
6000
4000
2000
0
-2000
9.5 9.0 8.5 8.0 7.5 7.0 6.5 6.0 5.5 5.0 4.5 4.0 3.5 3.0 2.5 2.0 1.5 1.0 0.5 0.0
f1 (ppm)

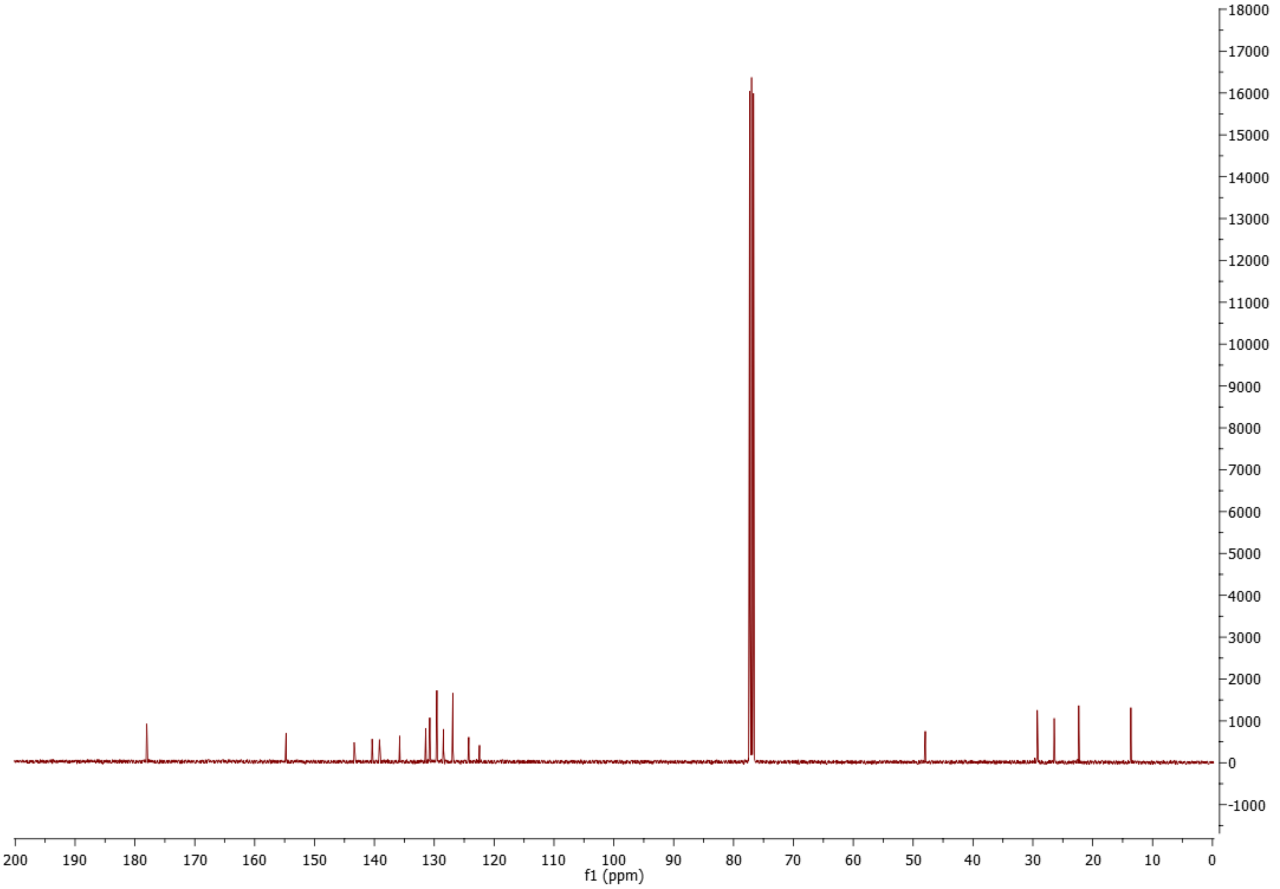
18000
17000
16000
15000
14000
13000
12000
11000
10000
9000
8000
7000
6000
5000
4000
3000
2000
1000
0
-1000
200 190 180 170 160 150 140 130 120 110 100 90 80 70 60 50 40 30 20 10 0
f1 (ppm)

Table S3. Data obtained from the mono- and two-dimensional NMR analysis of DP2, in deuterated chloroform ($CDCl_3$).

**Position**	**Residue**	**$^{13}C^{a}$**	**$^{1}H^{a}$, multiplicity (J in Hz)**	**^{1}H-^{1}H COSY**	**^{1}H-^{13}C HMBC**
2	C	154.64	-		
4	C	124.21	-		
5	C	124.42	-		
6	CH	177.77	9.72, s		124.42, 124.21
7	CH_2	25.69	2.66, t (8.0)	1.57	154.64, 28.82, 21.82
8	CH_2	28.82	1.57, quint (8.0)	2.66, 1.31	154.64, 12.56
9	CH_2	21.82	1.31, sest (8.0)	1.57, 0.87	25.69, 12.56
10	CH_3	12.56	0.87, t (7.5)	1.31	28.82, 21.82
11	CH_2	47.41	5.61, s		154.64, 124.42, 126.16
12	C	135.35	-		
13/17	CH	126.16	7.03, d (8.0)	7.08	139.37, 126.16, 4741
14/18	CH	129.24	7.08, d (8.0)	7.03	141.77, 135.35, 129.24
15	C	139.37	-		
18	C	141.77	-		
19	CH	130.52	7.49, d (7.0)	7.60	139.37, 127.48, 124.42
20	CH	130.26	7.60, t (7.1)	7.52, 7.49	141.77, 130.26
21	CH	127.48	7.52, t (7.1)	7.62, 7.60	130.52, 124.42
22	CH	130.26	7.62, d (7.0)	7.52	156.80, 141.77, 130.26
23	C	124.42	-		
24	C	156.80	-		

[a]Chemical shifts in ppm.

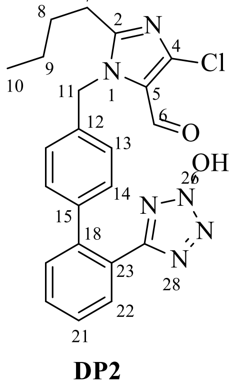

DP2

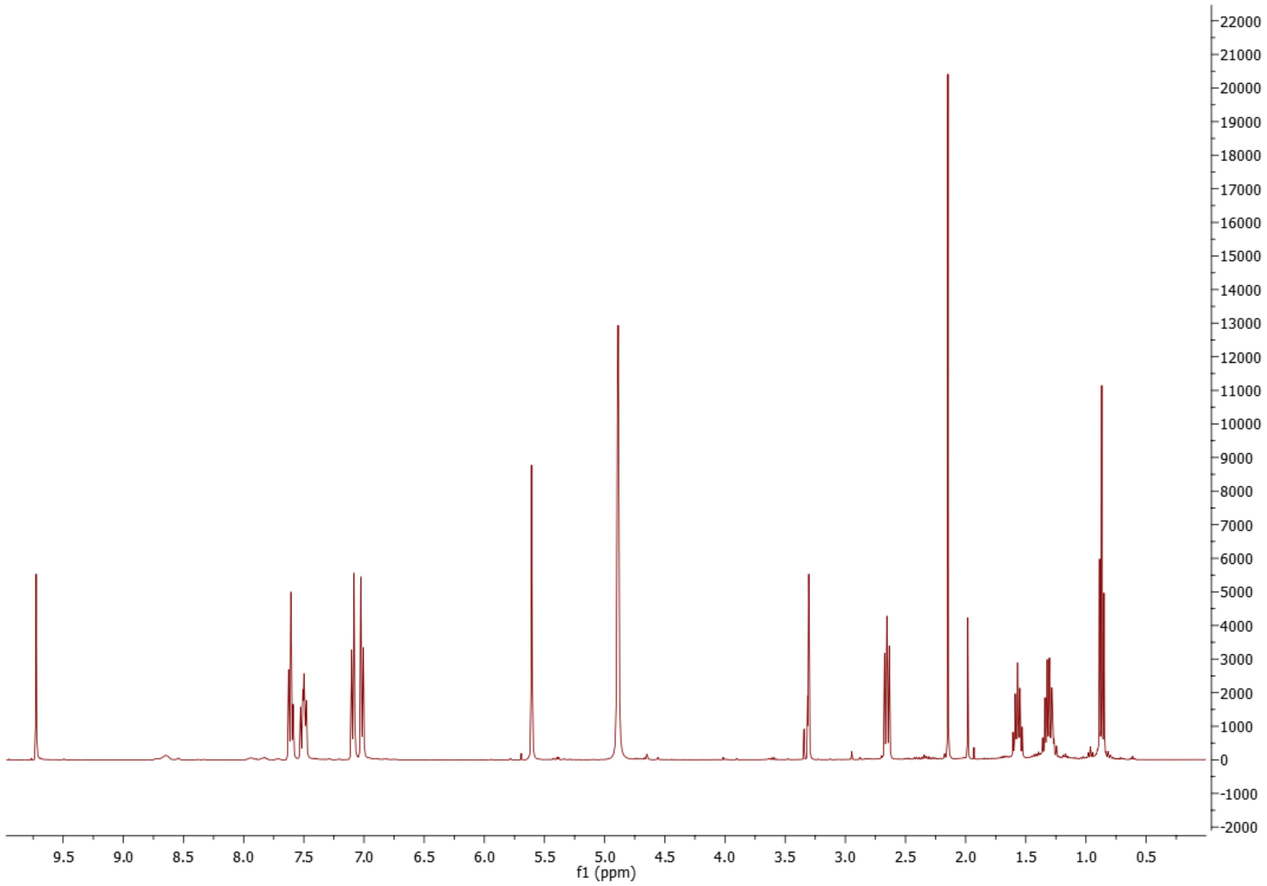
f1 (ppm)

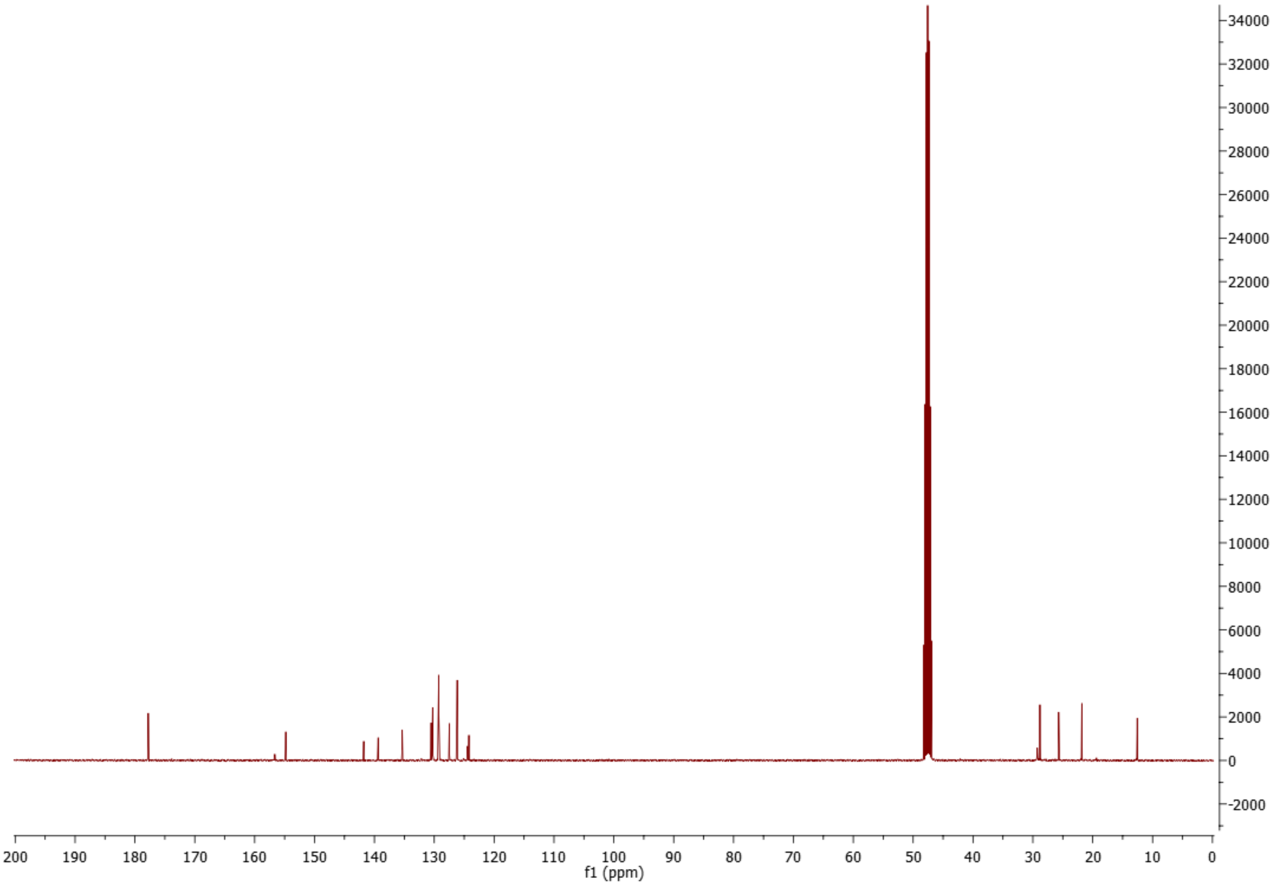
f1 (ppm)

Table S4. Data obtained from the mono- and two-dimensional NMR analysis of DP3, in deuterated methanol (CD_3OD).

**Position**	**Residue**	**$^{13}C^a$**	**$^1H^a$, multiplicity (J in Hz)**	**^1H-^1H COSY**	**^1H-^{13}C HMBC**
1	CH_3	19.97	2.01, s		175.40
2	C	175.40	-		
4	C	101.22	-		
5	CH_2	31.27	1.75, m<br>1.66, m	1.09, 0.75	101.22, 24.27, 21.54
6	CH_2	24.27	1.09, m<br>0.75, m	1.75, 1.66, 1.07, 0.87	101.22, 31.27, 21.54, 12.64
7	CH_2	21.54	1.07, m<br>0.87, m	1.09, 0.75, 0.65	31.27, 24.27, 12.64
8	CH_3	12.64	0.65, t (7.5)	1.07, 0.87	24.27, 21.54
10	C	160.55	-		
11	CH_2	42.41	4.96, overlapped<br>4.17, d (15.5)	4.17<br>4.96	160.55, 101.22, 128.13
12	C	135.30	-		
13/17	CH	128.13	7.32, d (8.0)	7.14	139.95, 128.13, 42.41
14/16	CH	129.15	7.14, d (8.0)	7.32	141.35, 135.30, 129.15
15	C	139.95	-		
18	C	141.35	-		
19	CH	130.45	7.48, d (7.0)	7.61	139.95, 127.18, 126.19
20	CH	129.92	7.61,m	7.52, 7.48	141.35, 131.1
21	CH	127.18	7.52, t (7.2)	7.62, 7.61	130.45, 126.19
22	CH	131.01	7.62, m	7.61, 7.52	158.35, 141.35, 129.92
23	C	126.19	-		
24	C	158.35	-		

[a]Chemical shifts in ppm.

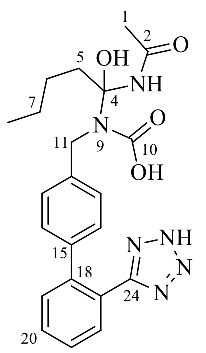

DP3

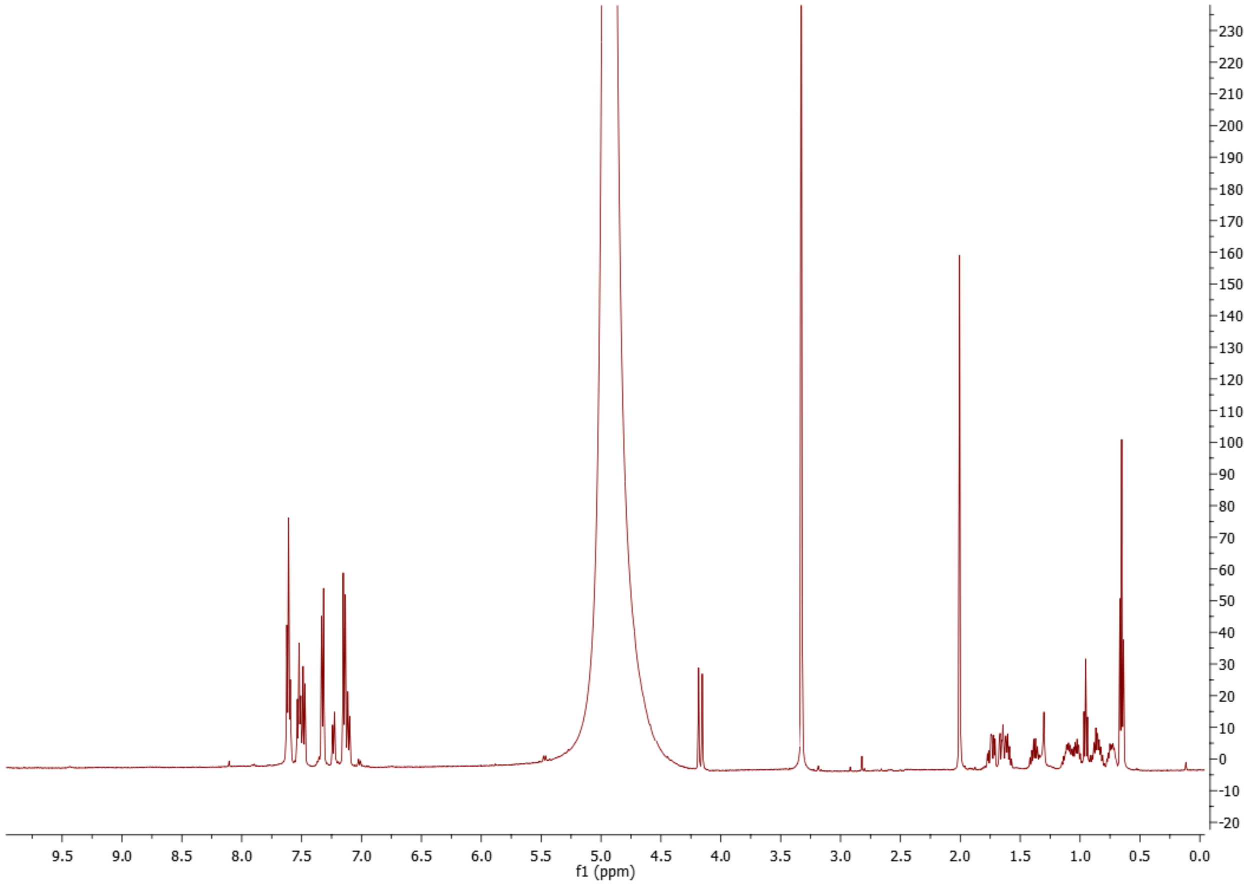
9.5 9.0 8.5 8.0 7.5 7.0 6.5 6.0 5.5 5.0 4.5 4.0 3.5 3.0 2.5 2.0 1.5 1.0 0.5 0.0
f1 (ppm)
230 220 210 200 190 180 170 160 150 140 130 120 110 100 90 80 70 60 50 40 30 20 10 0 -10 -20

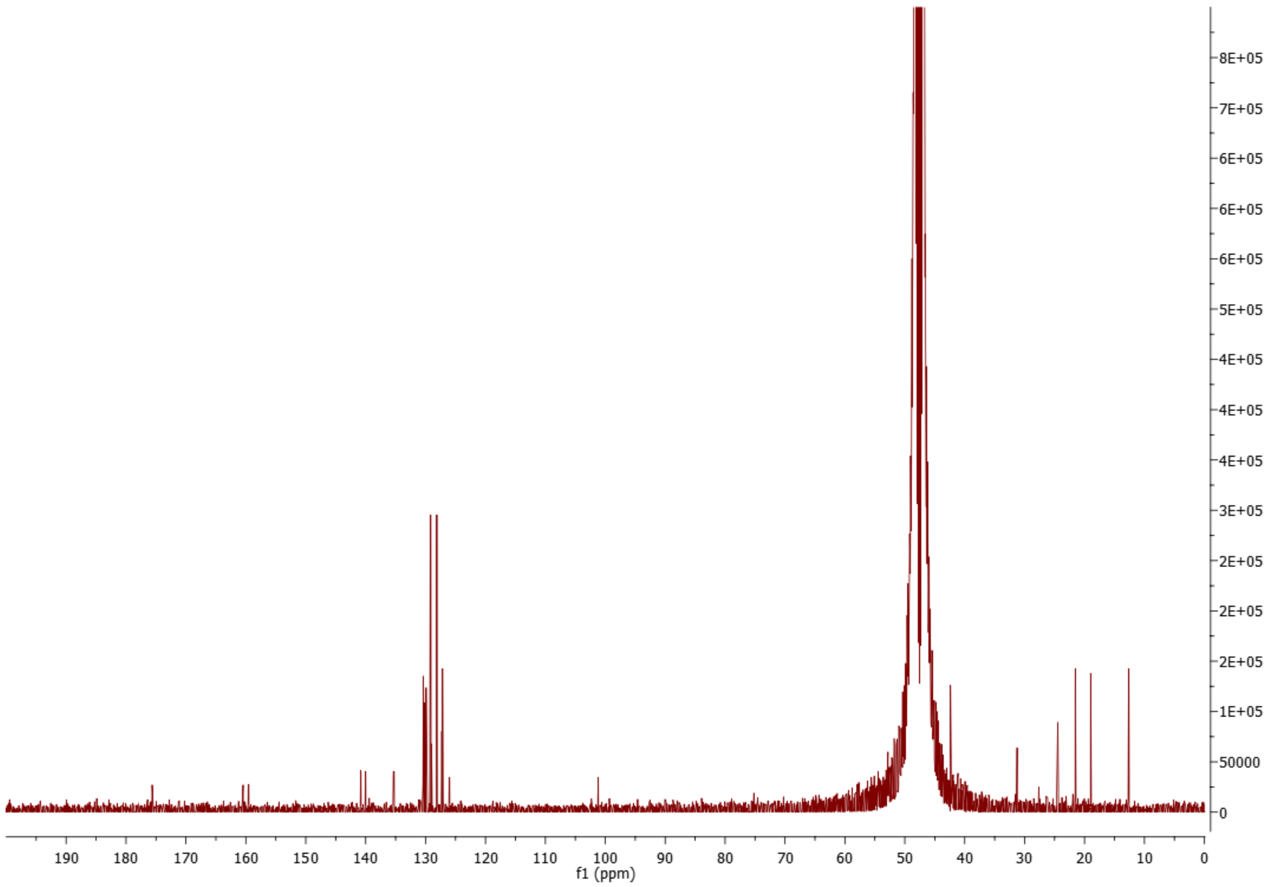
190 180 170 160 150 140 130 120 110 100 90 80 70 60 50 40 30 20 10 0
f1 (ppm)
8E+05 7E+05 6E+05 6E+05 6E+05 5E+05 4E+05 4E+05 4E+05 3E+05 2E+05 2E+05 2E+05 1E+05 50000 0

Table S5. Data obtained from the mono- and two-dimensional NMR analysis of DP4, in deuterated methanol (CD_3OD).

Position	Residue	$^{13}C^a$	$^1H^a$, multiplicity (J in Hz)	^1H-^1H COSY	^1H-^{13}C HMBC
1	C	168.35	-		
2	CH_2	32.39	2.48, t (7.9)	1.68	168.35, 29.19, 21.67
3	CH_2	29.19	1.68, quint (7.9)	2.48, 1.41	168.35, 32.39, 21.67, 12.42
4	CH_2	21.67	1.41, sest (7.5)	1.68, 0.97	32.39, 29.19, 12.42
5	CH_3	12.42	0.97, t (7.3)	1.41	29.19, 21.67
7	CH_2	45.48	4.41, s		168.35, 132.56127.16
8	C	132.56	-		
9/13	CH	127.16	7.19, d (8.4)	7.15	141.13, 127.16, 45.48
10/12	CH	129.53	7.15, d (8.4)	7.19	141.34, 132.56, 129.53
11	C	141.13	-		
14	C	141.34	-		
15	CH	129.92	7.44, d (7.7)	7.53	141.13, 128.33, 127.48
16	CH	128.91	7.53, t (7.8, 1.5)	7.46, 7.44	141.34, 130.55
17	CH	128.33	7.46, dt (7.6, 1.4)	7.57, 7.52	129.92, 127.48
18	CH	130.55	7.57, d (7.7)	7.52, 7.45	160.41, 141.34, 128.91
19	C	127.48	-		
20	C	160.41	-		

[a]Chemical shifts in ppm.

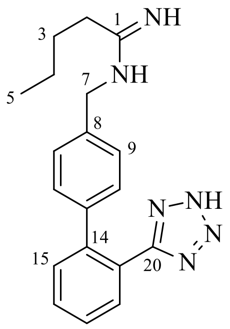

DP4

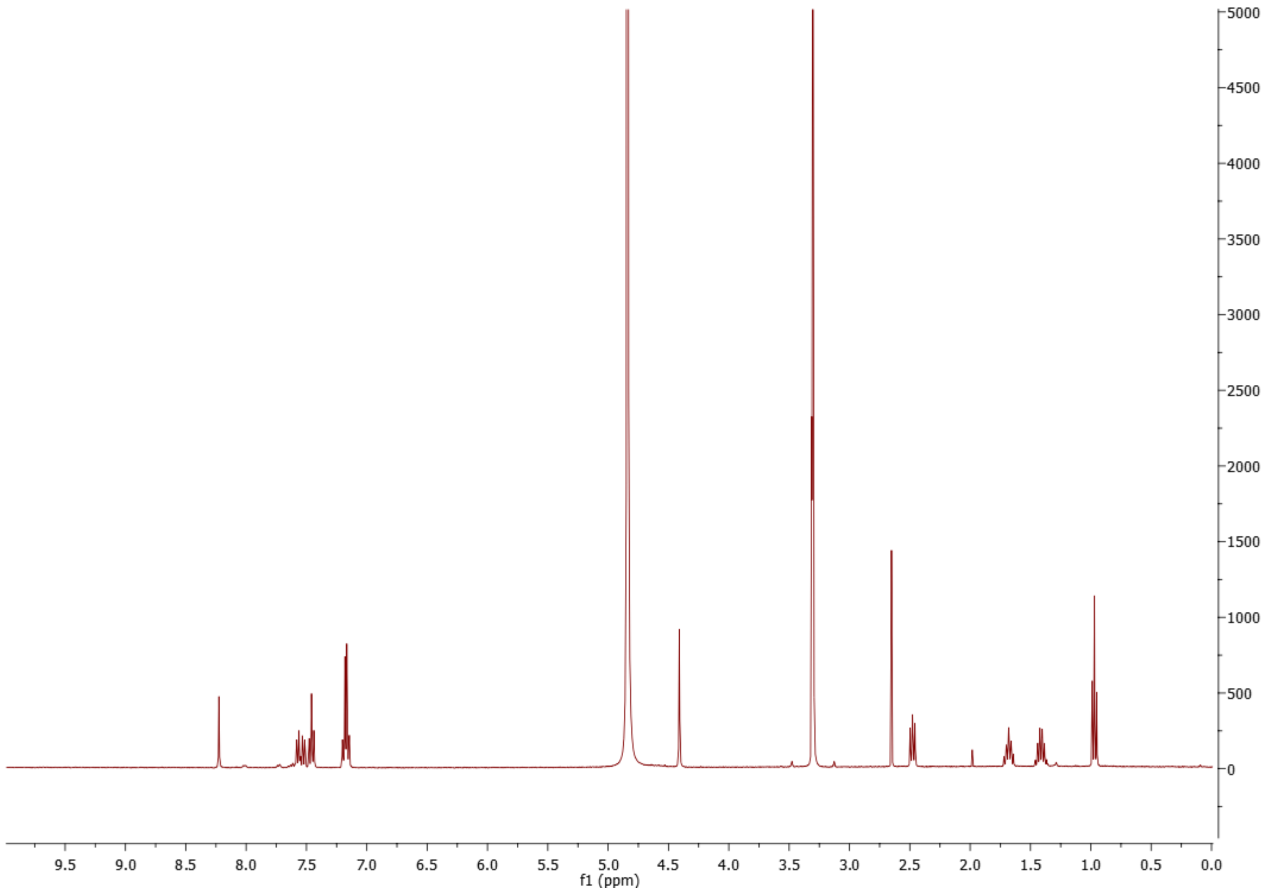

5000
4500
4000
3500
3000
2500
2000
1500
1000
500
0
9.5 9.0 8.5 8.0 7.5 7.0 6.5 6.0 5.5 5.0 4.5 4.0 3.5 3.0 2.5 2.0 1.5 1.0 0.5 0.0
f1 (ppm)

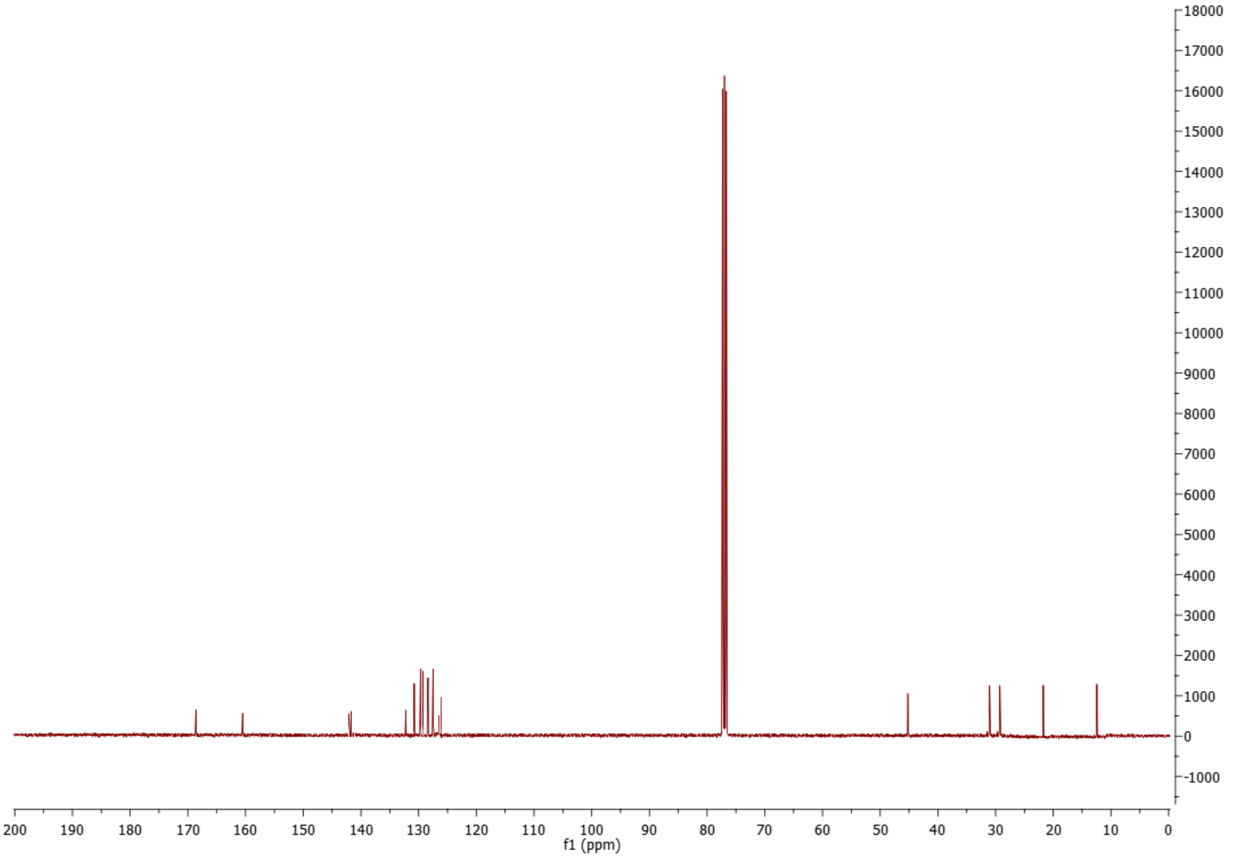

18000
17000
16000
15000
14000
13000
12000
11000
10000
9000
8000
7000
6000
5000
4000
3000
2000
1000
0
-1000
200 190 180 170 160 150 140 130 120 110 100 90 80 70 60 50 40 30 20 10 0
f1 (ppm)

Table S6. Data obtained from the mono- and two-dimensional NMR analysis of DP5, in deuterated dimethyl sulfoxide (CD_3SOCD_3).

Position	Residue	$^{13}C^a$	$^1H^a$, multiplicity (J in Hz)	^1H-^1H COSY	^1H-^{13}C HMBC
2	C	148.88	-		
4	C	127.16	-		
5	C	123.16	-		
6	CH_2	53.18	4.41, s		127.16, 123.16
7	CH_2	27.66	2.54, t (7.6)	1.48	148.88, 23.45
8	CH_2	30.88	1.48, quint (7.6)	2.54, 1.23	148.88, 15.41
9	CH_2	23.45	1.23, sest (7.6)	1.48, 0.72	27.66, 15.41
10	CH_3	15.41	0.72, t (7.6)	1.23	30.88, 23.45
11	CH_2	48.28	5.58, s		148.88, 127.75, 123.16, 116.56
12	C	141.86	-		
13	CH	116.56	8.30, s		127.75, 123.16, 48.28
14	C	131.42	-		
15	C	123.16	-		
16	CH	125.79	8.78, d (8.4)	7.48	141.86, 131.63, 131.42
17	CH	127.75	7.48, d (8.4)	8.78	123.16, 116.56, 48.28
18	C	131.63	-		
19	CH	125.84	8.73, d (8.0)	7.98	131.35, 123.16, 119.83
20	CH	134.24	7.98, t (8.0)	8.73, 7.85	131.63, 127.21
21	CH	131.35	7.85, t (8.1)	8.57, 7.98	125.84, 119.83
22	CH	127.21	8.57, d (8.0)	7.85	149.43, 134.24, 131.63
23	C	119.83	-		
24	C	149.43	-		

[a]Chemical shifts in ppm.

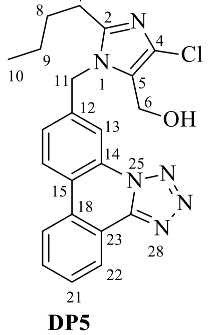

DP5

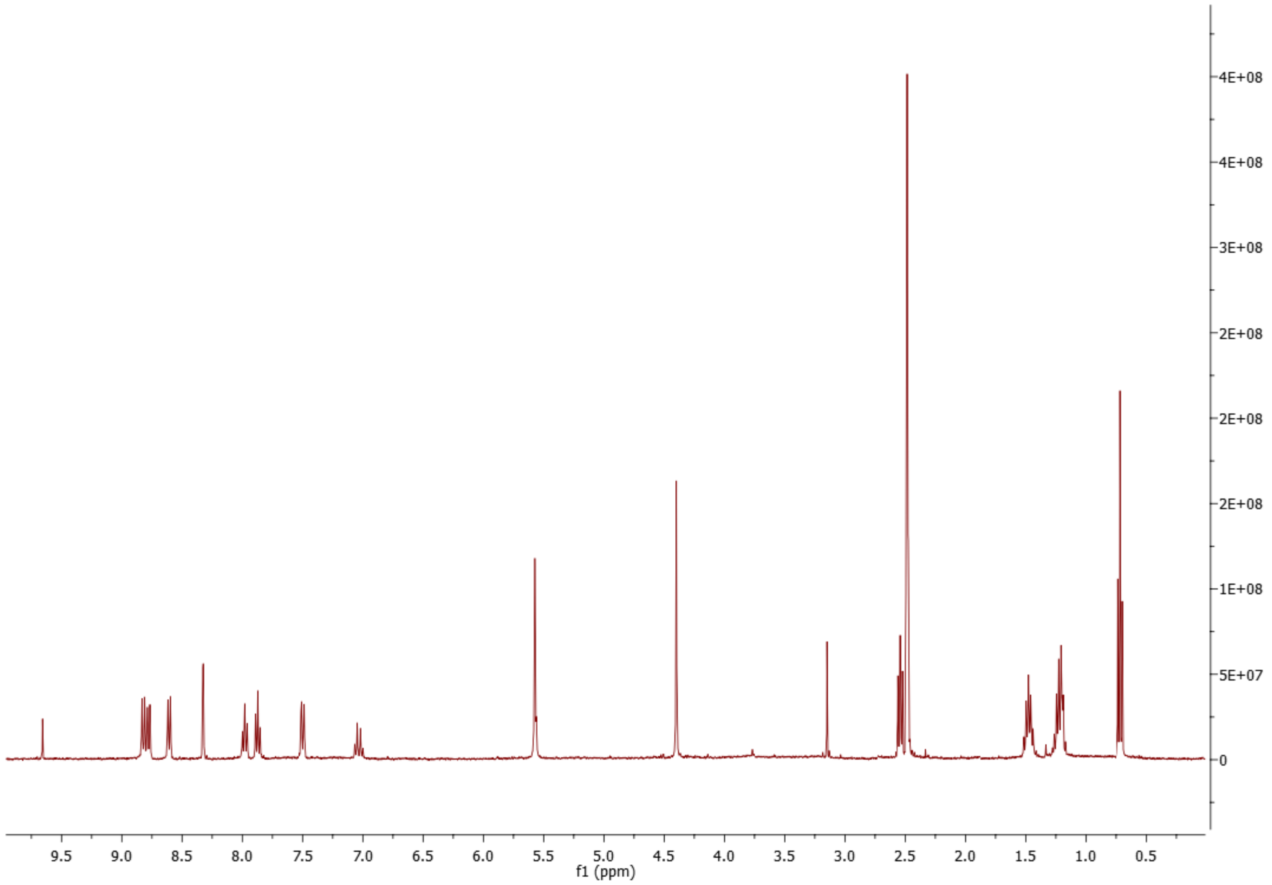
f1 (ppm)

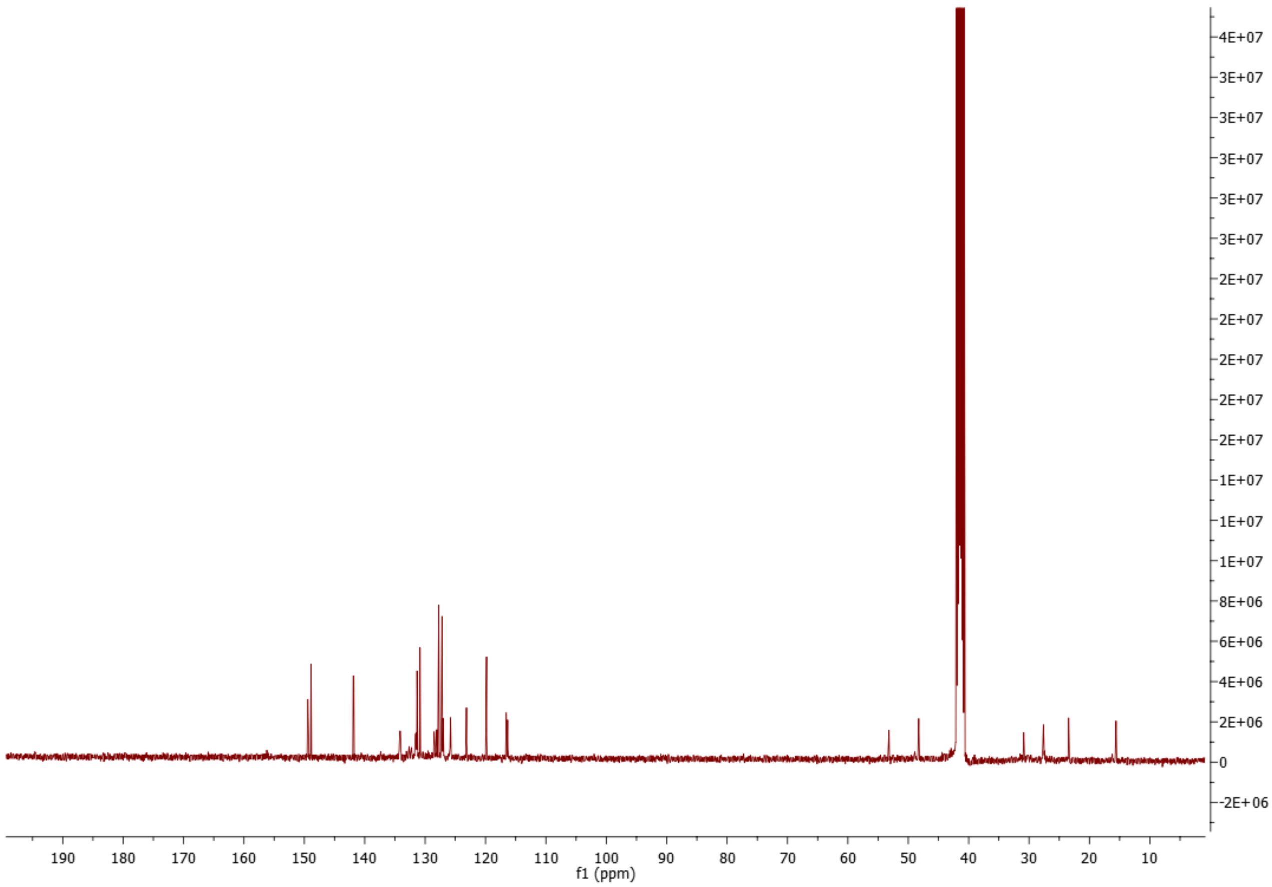
f1 (ppm)

Table S7. Data obtained from the mono- and two-dimensional NMR analysis of DP6, in deuterated chloroform ($CDCl_3$).

Position	Residue	$^{13}C^a$	$^1H^a$, multiplicity (J in Hz)	^1H-^1H COSY	^1H-^{13}C HMBC
2	C	154.67	-		
4	C	143.50	-		
5	C	124.39	-		
6	CH	178.09	9.81, s		143.50, 124.39
7	CH_2	29.31	2.76, t (6.0)	1.74	22.41
8	CH_2	26.59	1.74, quint (6.0)	2.76, 1.40	154.67, 13.67
9	CH_2	22.41	1.40, sest (6.0)	1.74, 0.90	29.31
10	CH_3	13.67	0.90, t (6.4)	1.40	26.59, 22.41
11	CH_2	47.94	5.83, s		154.67, 126.21, 124.39, 115.38
12	C	138.35	-		
13	CH	115.38	8.42, s		126.21, 122.08, 47.94
14	C	129.38	-		
15	C	122.08	-		
16	CH	124.96	8.49, d (6.8)	7.40	138.35, 129.58, 129.38
17	CH	126.21	7.40, d (6.8)	8.49	122.08, 115.38, 47.94
18	C	129.58	-		
19	CH	123.06	8.45, dd (6.8, 1.8)	7.91, 7.83	129.73, 122.08, 118.69
20	CH	132.16	7.91, t (6.9)	8.45, 7.83	129.58, 126.08
21	CH	129.73	7.83, t (6.8)	8.77, 7.91	123.06, 118.69
22	CH	126.08	8.77, dd (6.8, 1.8)	7.91, 7.83	147.38, 132.16, 129.58
23	C	118.69	-		
24	C	147.38	-		

[a]Chemical shifts in ppm.

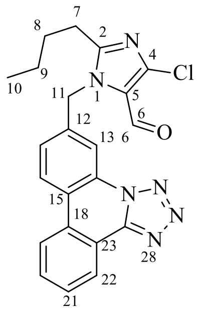

DP6

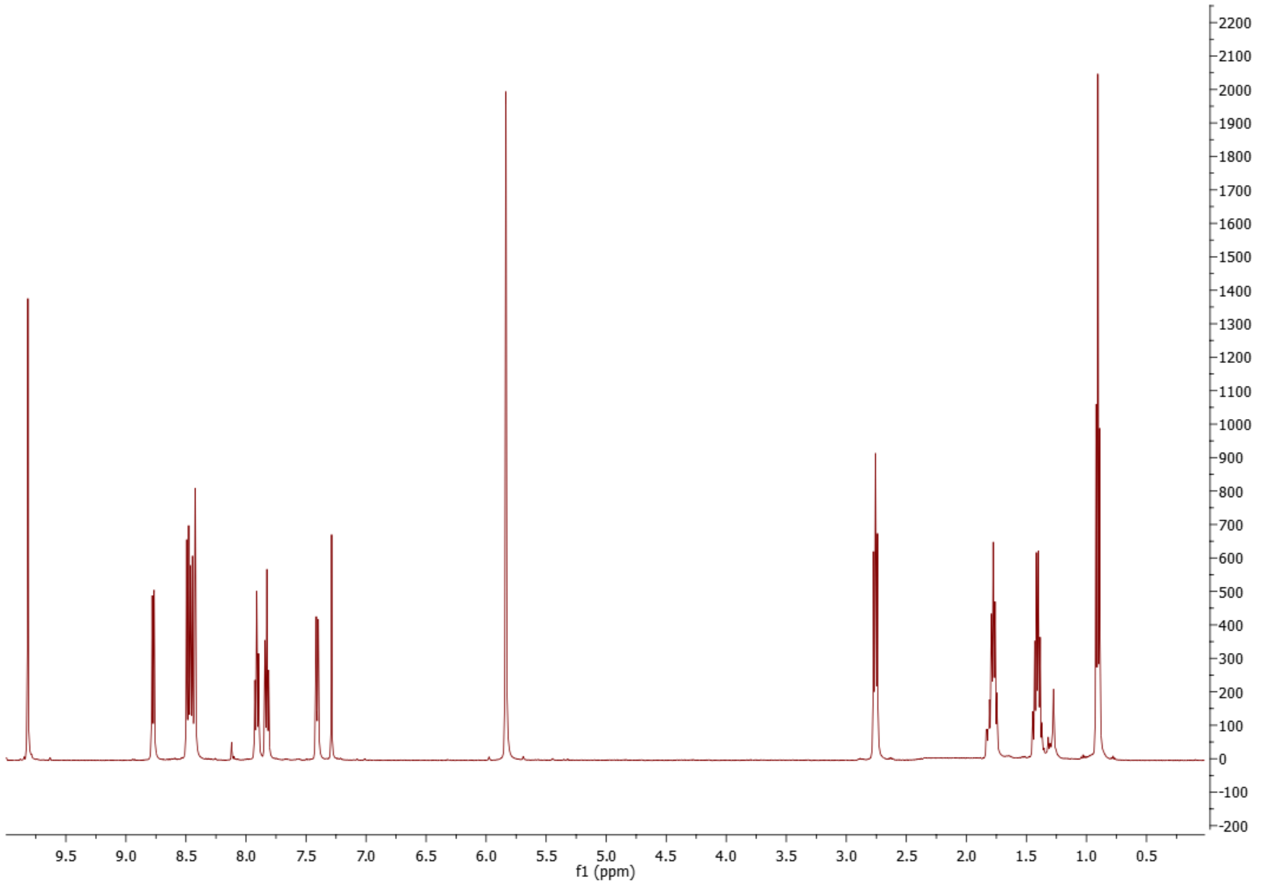

2200 2100 2000 1900 1800 1700 1600 1500 1400 1300 1200 1100 1000 900 800 700 600 500 400 300 200 100 0 -100 -200
9.5 9.0 8.5 8.0 7.5 7.0 6.5 6.0 5.5 5.0 4.5 4.0 3.5 3.0 2.5 2.0 1.5 1.0 0.5
f1 (ppm)

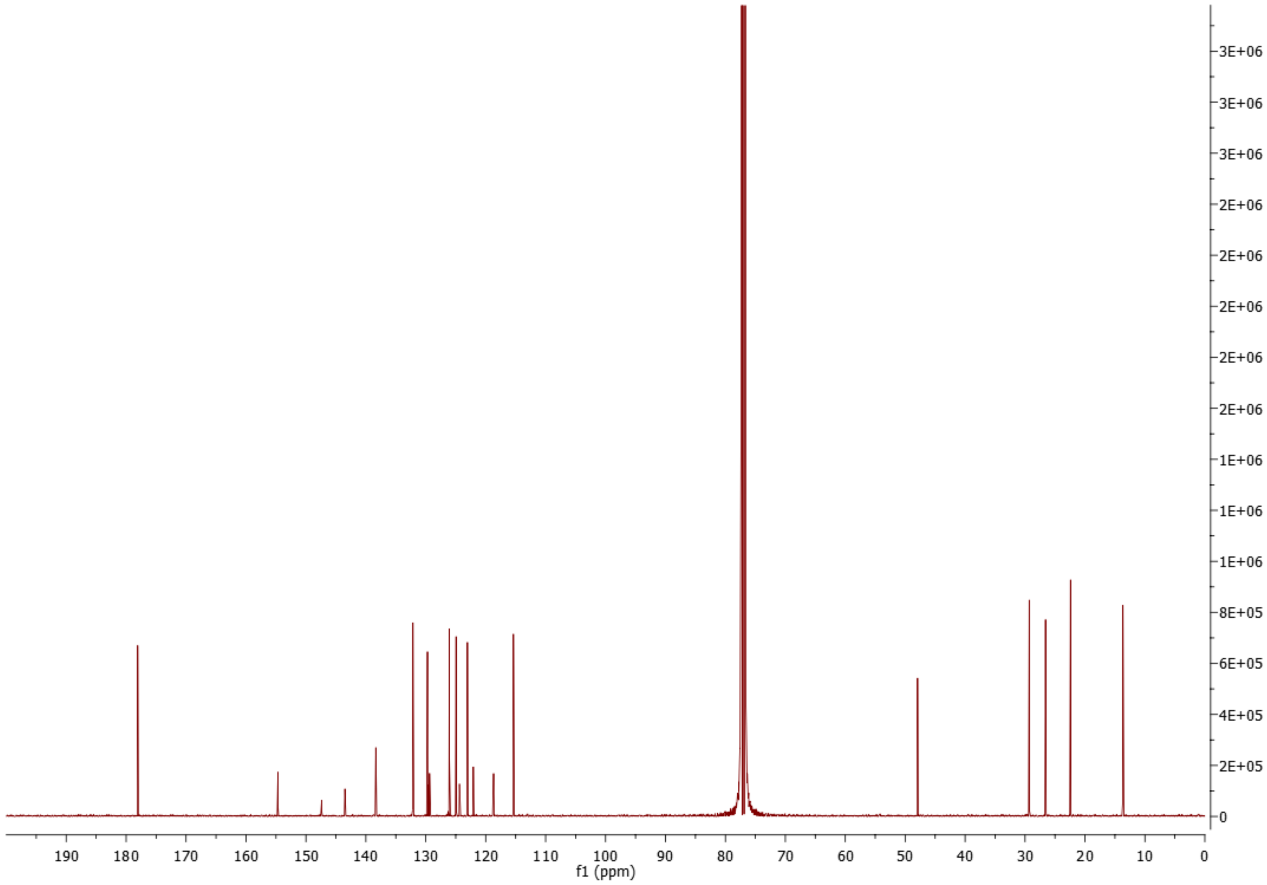

3E+06 3E+06 3E+06 2E+06 2E+06 2E+06 2E+06 2E+06 1E+06 1E+06 1E+06 8E+05 6E+05 4E+05 2E+05 0
190 180 170 160 150 140 130 120 110 100 90 80 70 60 50 40 30 20 10 0
f1 (ppm)

Table S8. Data obtained from the mono- and two-dimensional NMR analysis of DP7, in deuterated dimethyl sulfoxide (CD_3SOCD_3).

Position	Residue	$^{13}C^a$	$^1H^a$, multiplicity (J in Hz)	^1H-^1H COSY	^1H-^{13}C HMBC
2	C	155.07	-		
4	C	141.71	-		
5	C	125.86	-		
6	CH	178.50	9.71, s		141.71, 125.86
7	CH_2	29.09	2.75, t (7.2)	1.57, 1.27	155.07, 25.87, 22.02
8	CH_2	25.87	1.57, quint (7.2)	2.75, 1.27	155.07, 29.09, 22.02, 13.94
9	CH_2	22.02	1.27, sest (7.2)	1.57, 0.77	29.09, 25.87, 13.94
10	CH_3	13.94	0.77, t (7.2)	1.57, 1.27	25.87, 22.02
11	CH_2	47.40	5.89, s		155.07, 126.46, 125.86, 114.84
12	C	139.60	-		
13	CH	114.84	8.31, d (1.6)	8.82	126.46, 121.91, 47.40
14	C	129.45	-		
15	C	121.91	-		
16	CH	126.34	8.82, d (8.4)	8.31, 7.50	129.51, 129.45, 139.60
17	CH	126.46	7.50, dd (8.4, 1.6)	8.82	121.91, 114.84, 47.40
18	C	129.51	-		
19	CH	124.64	8.78, d (8.8)	7.99, 7.88	129.81, 121.91, 118.49
20	CH	132.85	7.99, t (8.5)	8.78, 7.88	129.51, 126.22
21	CH	129.81	7.88, t (8.5)	8.63, 7.99	124.64, 118.49
22	CH	126.22	8.63, d (8.4)	7.99, 7.88	147.49, 132.85, 129.51
23	C	118.49	-		
24	C	147.49	-		

[a]Chemical shifts in ppm.

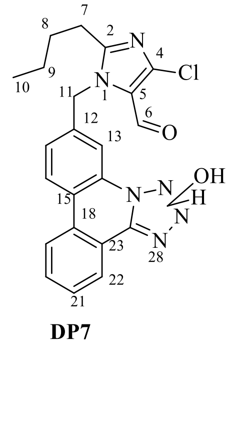

DP7

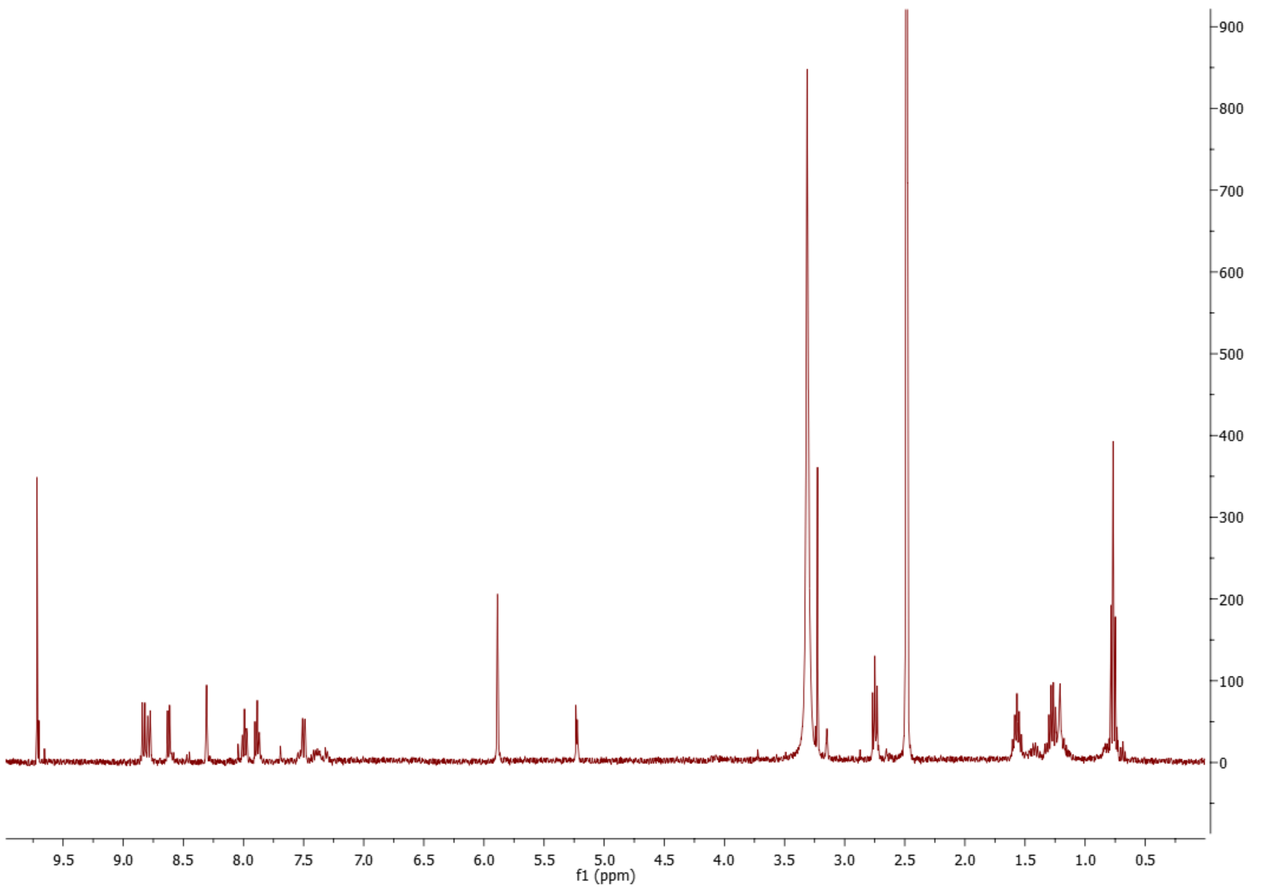

900
800
700
600
500
400
300
200
100
0
9.5 9.0 8.5 8.0 7.5 7.0 6.5 6.0 5.5 5.0 4.5 4.0 3.5 3.0 2.5 2.0 1.5 1.0 0.5
f1 (ppm)

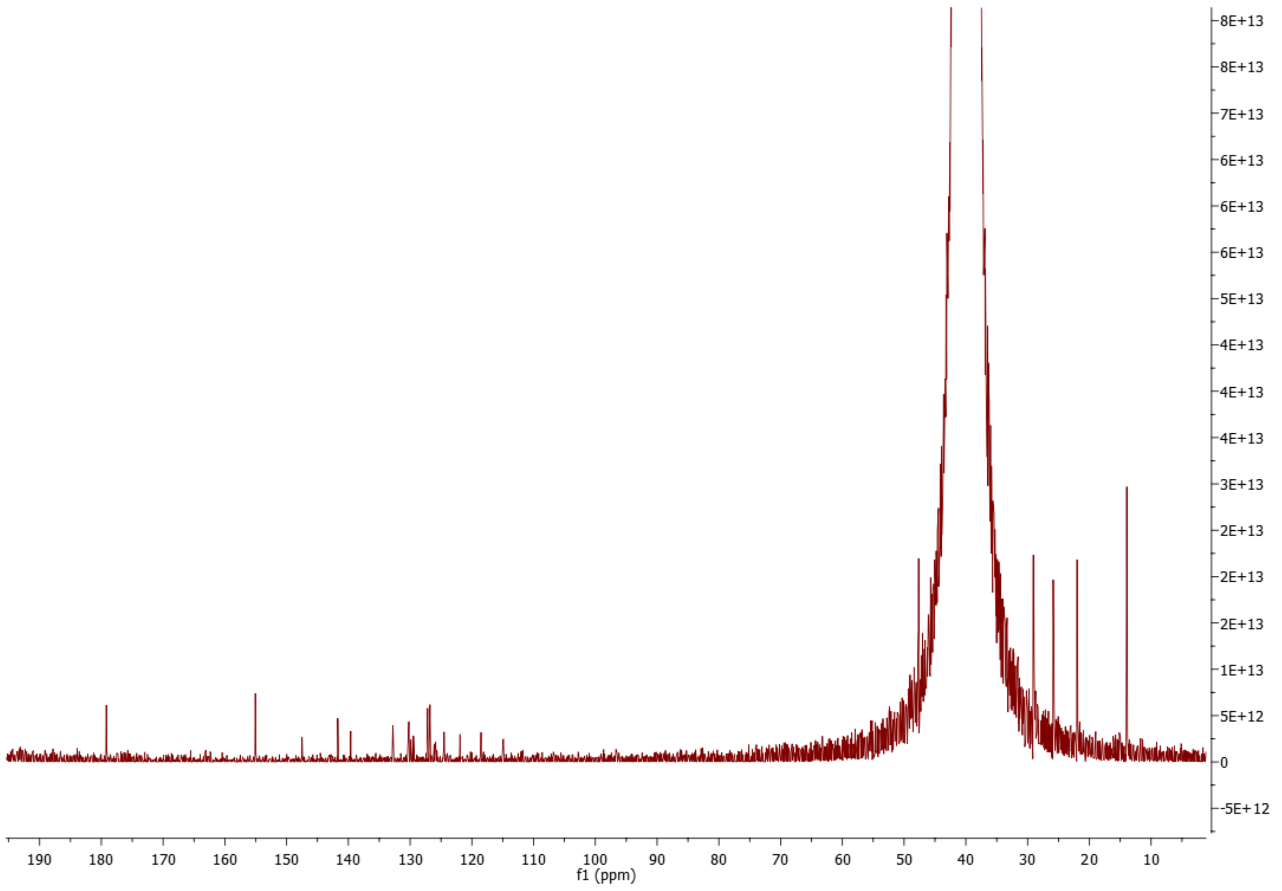

8E+13
8E+13
7E+13
6E+13
6E+13
6E+13
5E+13
4E+13
4E+13
4E+13
3E+13
2E+13
2E+13
2E+13
1E+13
5E+12
0
-5E+12
190 180 170 160 150 140 130 120 110 100 90 80 70 60 50 40 30 20 10
f1 (ppm)

Table S9. Data obtained from the mono- and two-dimensional NMR analysis of DP8, in deuterated chloroform ($CDCl_3$).

Position	Residue	$^{13}C^{a}$	$^{1}H^{a}$, multiplicity (J in Hz)	^{1}H-^{1}H COSY	^{1}H-^{13}C HMBC
2	C	156.41	-		
4	C	127.50	-		
5	C	124.44	-		
6	C	161.25	-		
7	CH_2	25.50	2.33, t (7.5)	1.63	22.05
8	CH_2	29.67	1.63, quint (7.5)	2.33, 1.35	156.41, 14.09
9	CH_2	22.05	1.35, sest (7.5)	1.63, 0.87	25.50
10	CH_3	14.09	0.87, t (7.5)	1.35	29.67, 22.05
11	CH_2	43.35	4.83, s		156.41, 124.44, 127.73, 116.79
12	C	139.60	-		
13	CH	116.79	8.62, s		127.73, 121.89, 43.35
14	C	129.50	-		
15	C	121.89	-		
16	CH	125.00	8.52, d (8.0)	7.72	139.60, 129.50, 129.47
17	CH	127.73	7.72, d (8.0)	8.52	121.89, 116.79, 43.35
18	C	129.47	-		
19	CH	123.12	8.50, dd (7.9, 1.4)	7.92, 7.83	129.50, 121.89, 118.57
20	CH	132.31	7.92, t (8.0)	8.50, 7.83	129.47, 126.25
21	CH	129.50	7.83, t (8.1)	8.79, 7.92	123.12, 118.57
22	CH	126.25	8.79, dd (7.9, 1.5)	7.92, 7.83	147.46, 132.31, 129.47
23	C	118.57	-		
24	C	147.46	-		
OCH_3	CH_3	53.88	3.96, s		161.25

[a]Chemical shifts in ppm.

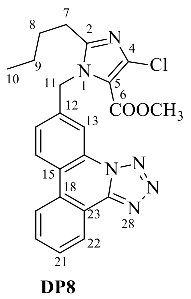

DP8

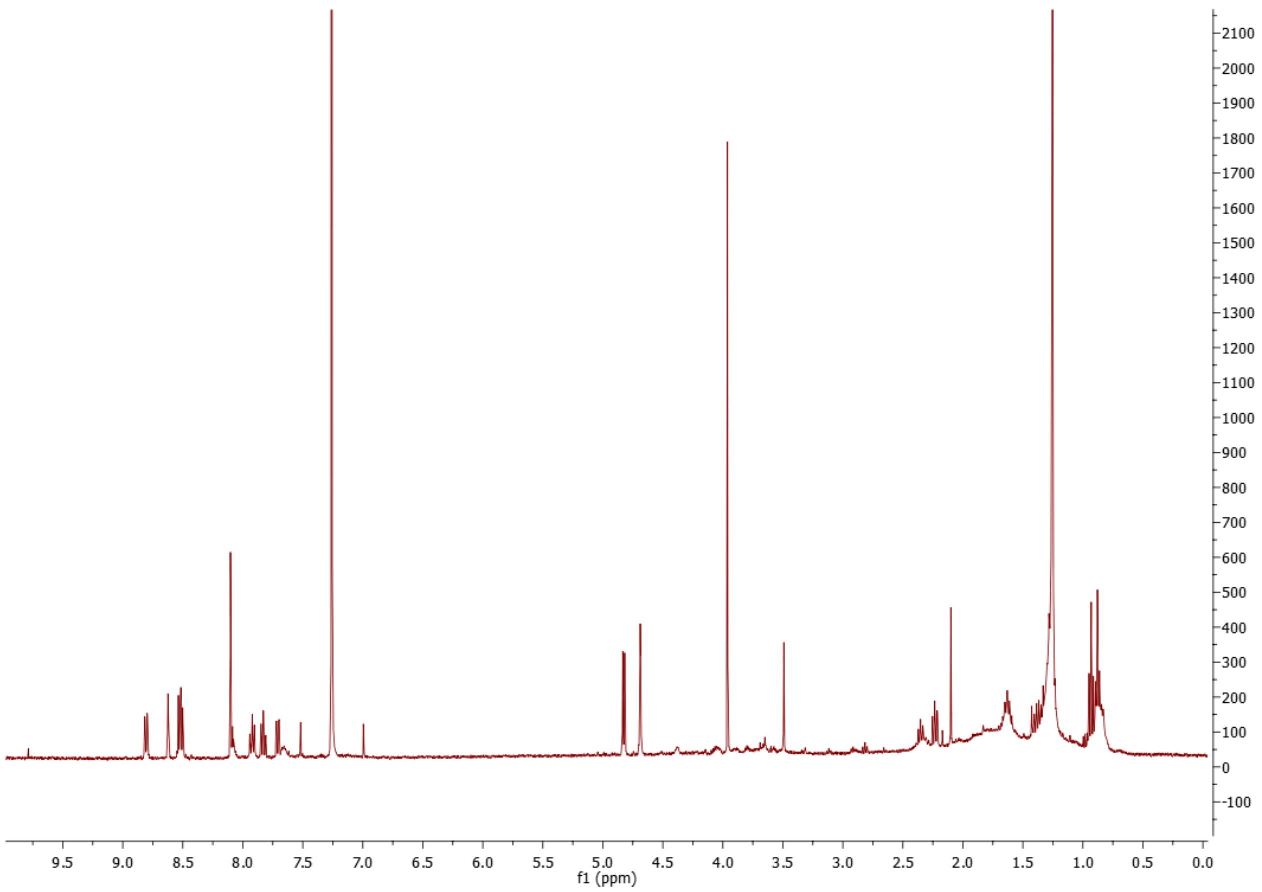
f1 (ppm)

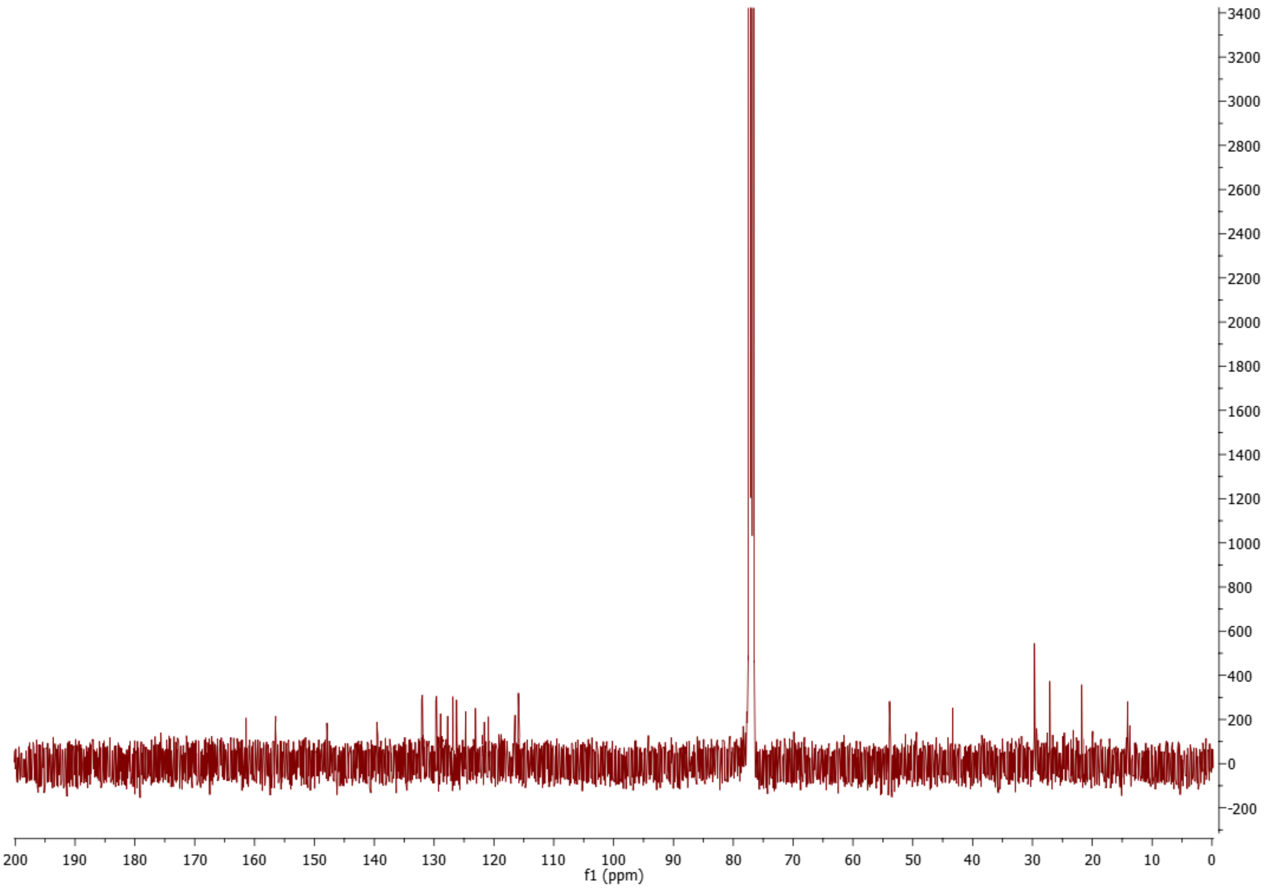
f1 (ppm)

Table S10. Data obtained from the mono- and two-dimensional NMR analysis of DP9, in deuterated methanol (CD_3OD).

Position	Residue	$^{13}C^a$	$^1H^a$, multiplicity (J in Hz)	^1H-^1H COSY	^1H-^{13}C HMBC
1	C	170.20	-		
2	CH_2	34.92	2.61, t (7.6)	1.78	170.20, 30.51, 22.50
3	CH_2	30.51	1.78, quint (8.0)	2.61, 1.49	170.20, 34.92, 22.50, 13.10
4	CH_2	22.50	1.49, sest (7.7)	1.78, 1.02	34.92, 30.51, 13.10
5	CH_3	13.10	1.02, t (7.4)	1.49	30.51, 22.50,
7	CH_2	46.28	4.60, s		170.20, 128.74, 115.85
8	C	138.87	-		
9	CH	115.85	8.68, d (1.2)	8.84	128.74, 123.59, 130.80, 46.28
10	C	130.80			
11	C	123.59			
12	CH	126.55	8.84, d (8.6)	8.68, 7.82	138.87, 130.80, 123.59
13	CH	128.74	7.82, dd (8.6, 1.2)	8.84	123.59, 115.85, 46.28
14	C	130.80	-		
15	CH	124.80	8.78, dd (8.8, 1.0)	8.02	130.86, 123.59, 119.54
16	CH	133.56	8.02, dt (8.8, 1.2)	8.78, 7.91	130.80, 126.45
17	CH	130.86	7.91, t (8.2, 1.0)	8.73, 8.02	124.80, 119.54
18	CH	126.45	8.73, d (7.7, 1.2)	7.91	148.47, 133.56, 130.80
19	C	119.54	-		
20	C	148.47	-		

[a]Chemical shifts in ppm.

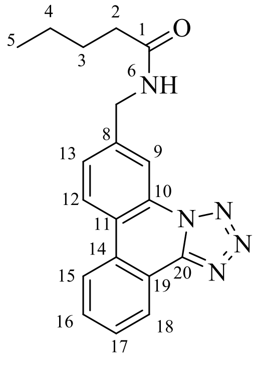

DP9

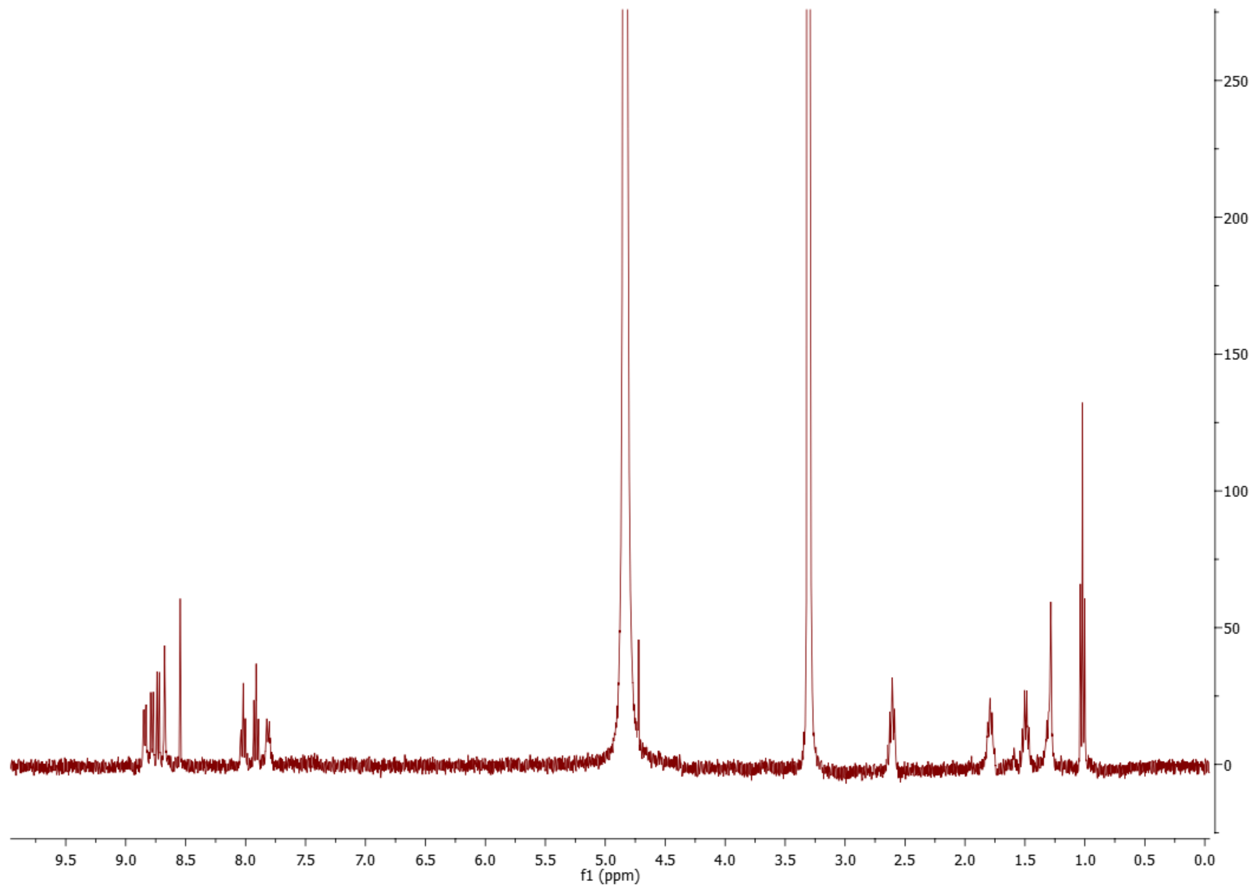

f1 (ppm)

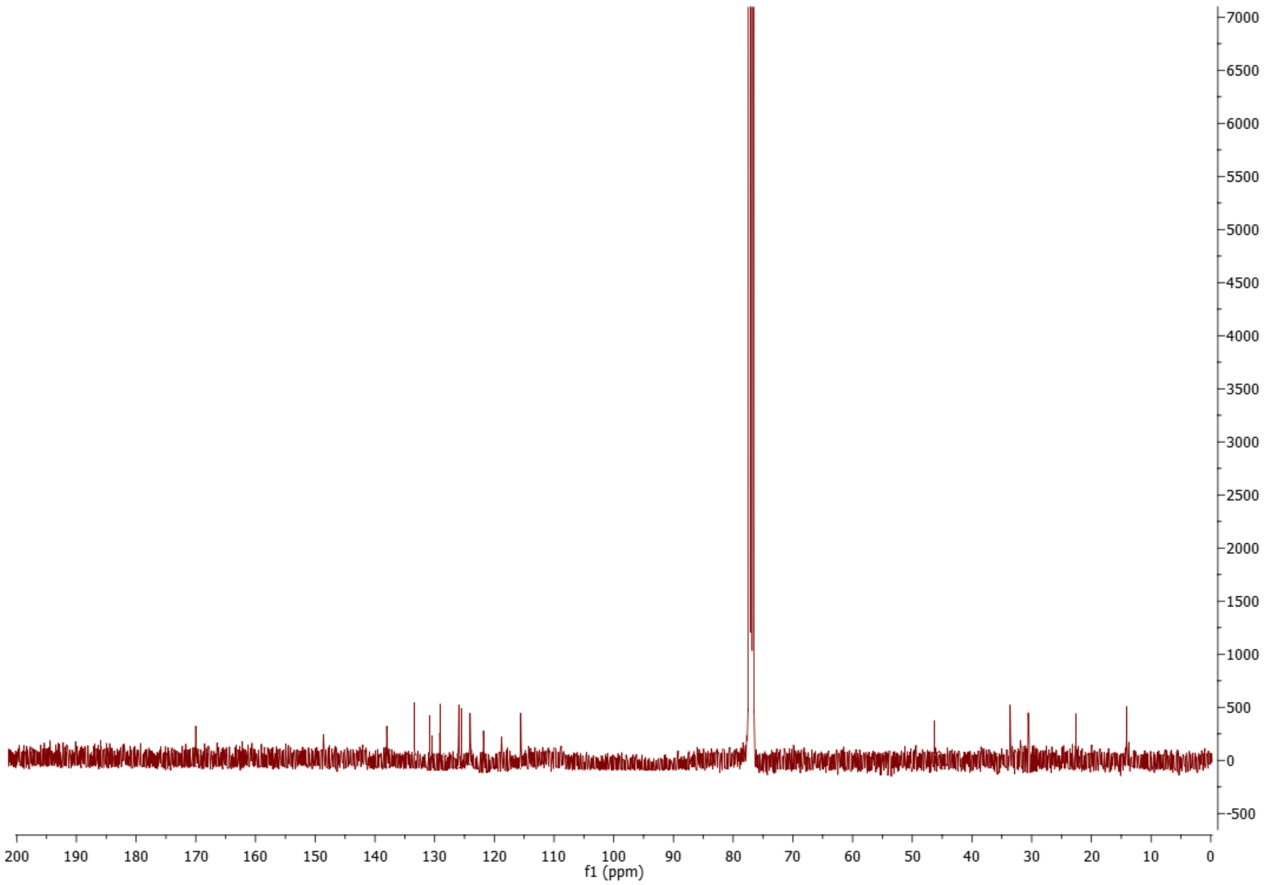

f1 (ppm)
